# Essential and Potentially Toxic Elements in Commercial Milk Formulas: Health Risk Assessment Through a Systematic Review and Meta-analysis

**DOI:** 10.1007/s12011-026-05088-4

**Published:** 2026-04-09

**Authors:** Caroline Bekman Diniz Largueza, Cinthia de Carvalho Couto, Michel Carlos Mocellin, Fernando Lamarca, Tatiana Dillenburg Saint’Pierre, Otniel Freitas Silva, Simone Augusta Ribas

**Affiliations:** 1 Food and Nutrition Graduate Program (PPGAN), Nutrition School, Federal University of the State of Rio de Janeiro (UNIRIO), Avenida Pasteur, 296 - Urca, Rio de Janeiro, Brazil; 2 Fundamental Nutrition Department, Nutrition School, Federal University of the State of Rio de Janeiro (UNIRIO), Avenida Pasteur, 296 - Urca, Rio de Janeiro, Brazil; 3 Department of Applied Nutrition, Institute of Nutrition, Rio de Janeiro State University (Uerj), Rua São Francisco Xavier, 524, Rio de Janeiro, CEP: 20559-900 Brazil; 4 Chemistry Departament, Pontifical Catholic University of Rio de Janeiro (PUC- Rio), Rua Marquês de São Vicente, 225 - Gávea, Rio de Janeiro, RJ CEP: 22451- 900 Brazil; 5 Embrapa Agroindústria de Alimentos, Av. das Américas 29501, Rio de Janeiro, RJ 23020-470, CEP 23020-470 Brazil; 6 Food and Nutrition Graduate Program (PPGAN), Nutrition and Public Health Department, Nutrition School, Federal University of the State of Rio de Janeiro (UNIRIO), Avenida Pasteur, 296 - Urca, Rio de Janeiro, Brazil

**Keywords:** Infant formula, Potentially toxic element, Risk assessment, commercial milk formulas, Food analysis

## Abstract

Although commercial milk formulas (CMF) provide essential microelements for child development, they may also contain potentially toxic elements (PTE) that pose health risks depending on their concentration. This systematic review and meta-analysis investigated the presence of essential and toxic elements in CMF. Observational studies assessing element concentrations in three types of CMF (infant formulas [IF], follow-up formulas for older infants [FFI], and products for young children [PYC]) were retrieved from Embase, Scopus, PubMed, Web of Science, Livivo, and Lilacs. Health risk assessment was conducted using the target hazard quotient (THQ). Thirty studies were included in the qualitative synthesis, and eighteen in the meta-analysis. The highest concentrations across CMF types were observed for Al, Fe, Mn and Ni. By WHO regions, the Eastern Mediterranean and the Americas showed the highest levels in IF and FFI, respectively, mainly for Al (IF: 4600 µg kg⁻¹; FFI: 838 µg kg⁻¹), Cu (IF: 2420 µg kg⁻¹; FFI: 2127.23 µg kg⁻¹), and Mn (IF: 984 µg kg⁻¹; FFI: 1853.30 µg kg⁻¹). For PYC, the highest contents were found in the Americas (Al: 1522.47 µg kg⁻¹) and Europe (Mn: 1160 µg kg⁻¹). Risk assessment indicated that daily consumption of IF may be unsafe due to As, Cd, Cu, Co, Fe, methyl-Hg, and Zn. Similar risks were identified in FFI for Cu, Fe, Ni, Se and Zn, and in PYC for Ni. The sum of THQ values exceeded 1 in several countries for all CMF types, suggesting potential non-carcinogenic risks. Continuous monitoring of essential and toxic elements in CMF is necessary to safeguard children’s health.

**Graphical Abstract:**

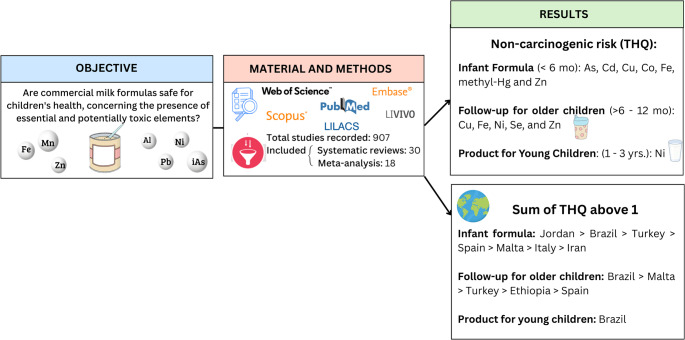

**Supplementary Information:**

The online version contains supplementary material available at 10.1007/s12011-026-05088-4.

## Introduction

Increasing continued breastfeeding rates is still a challenge in most countries [1], since babies under 6 months should be exclusively breastfed and then continued until 2 years or more [2, 3]. Simultaneously, commercial milk formula (CMF) is increasingly present in the diet of babies and children, often fully replacing breastfeeding [4]. Across different countries, the consumption of these products is driven by geographical location, cultural traditions, market influence, and economic conditions [5].

However, acquiring these products can represent an additional financial cost compared to breastfeeding and traditional cow’s milk, especially for families with low purchasing power, burdening the human and economic costs of the health system [6], making it essential to understand whether they are necessary and important in children’s diets.

Meanwhile, the importance of milk and its derivatives in the diet is also attributed to their content of essential nutrients for growth and development during the preschool years, which is why their daily consumption is recommended to meet nutritional requirements for protein, calcium, and phosphorus [7]. Against this backdrop, over the past decade, the food industry has intensified the commercialization of milk-based dairy products for young children, considering this a highly profitable sector.

Unlike infant formulas aimed at infants, CMF for children over 1 year old are described in the literature under many different terminologies [8]. In addition, the similarity of the label between these products favours cross-promotion, which makes it difficult for consumers to choose at the time of purchase and for health professionals to recommend appropriate use [9].

Faced with this new market scenario, in 2023 the Codex Alimentarius published an updated classification of infant formula categories for infant feeding, which are called: (a) infant formulas (IF) for infants between 0 and 6 months of age, (b) follow-up formula for older infants (FFI) (from 6 months of age), when food introduction begins, (c) product for young children (PYC) for children over 1 year of age [2, 3]. As Brazil is a signatory to the Codex, this study will adopt its classification and the general term CMF when referring to all of them.

In terms of minerals, milk and its derivatives contain more than twenty essential minerals, which have metabolic functions in human health [10]. However, industrially processed foods can also contain non-essential minerals as contaminants, which can be harmful even in small concentrations. These elements are also known as potentially toxic elements (PTE), such as Al, As, Cd, Hg, Pb and U. The level of toxicity of elements, for instance, can vary according to the duration and route of exposure, the form or species of the element, and also the genetic characteristics of each person [10–12] When ingested in certain concentrations, they can lead to gastrointestinal, renal, and hepatic diseases, as metabolic, mental, hormonal, and immunological disorders, among others [13–16] The effects of exposure to PTE put young children as a vulnerable group, since contaminant limits recommended for adults cannot be adopted for infants, as their diet, energy requirements, and nutrient intake are distinct [17].

Given this context, many countries have established different types of guidelines to monitor the levels of toxic elements in food products, with a view to their safety for human consumption [18, 19]. The Codex Alimentarius sets maximum limits for most contaminants. It is important to note that many of them are naturally present in the environment, so it would not be possible to set a limit equal to zero [1].

Even though infant formulas are monitored and controlled in most countries [4, 17], it is noteworthy that there are still a few studies in the literature that present a detailed composition of essential and potentially toxic elements in most dairy products aimed at early childhood [20–23]. Therefore, this systematic review and meta-analysis aimed to investigate the essential and PTE concentrations in CMF (IF, FFI, and PYC) intended for children and estimate the risk to their health status.

## Materials and Methods

### Protocol and Registration

This study consists of a systematic review with meta-analysis. The structure of this review followed the Preferred Reporting Items for Systematic Reviews and Meta-Analyses (PRISMA) guidelines [24]. The PRISMA checklist is provided in Supplementary Material (Table S1). To ensure transparency and minimize biases during its execution, the protocol was publicly registered on Open Science Framework on June 14, 2022 (10.17605/OSF.IO/2YNKB).

### Research Question

The research question was formulated using the Population, Exposure, Comparison, and Outcome (PECO) strategy (Table S2) as follows: Do CMFs contain levels of potentially toxic elements that could harm children’s health?

### Eligibility Criteria

#### Inclusion Criteria

This review included observational studies that investigated the concentration of some essential and PTE, expressed in mass of the element for mass of the CMF (mg kg^−1^or µg kg^− 1^), in the three types of CMF (IF, FFI, and PYC).

The search included studies published in the last seven years to ensure that the evidence reflects the current market landscape, including the discontinuation of older products and the launch of new commercial formulations. The search was not restricted by language, publication status, or country to minimize publication and retrieval bias.

#### Exclusion Criteria

The following studies were excluded: (a) those including non-fortified milk from cow or other animals (e.g., goat, buffalo, among others), solid or fermented milk products (e.g., yogurt, cheese in general); (b) those focusing on PTE measurement methods (development, optimization, or validation); and studies related to PTE levels in dairy products that were obtained under abnormal conditions (e.g. environmental disasters); (c) review studies, conference abstract, personal opinions, books, case report, research protocols, clinical trials, and qualitative studies, in vivo (animal) and in vitro experimental studies.

### Information Sources and Search Strategy

The literature search was conducted in Embase, Scopus, PubMed, Web of Science, Livivo, and Lilacs databases using keyword matching, boolean operators, and search facilitators (citations, truncated words, proximity operators) adapted for each database (Table S3) by two researchers. The search was conducted on February 20, 2024. The reference lists of the included studies were also searched to identify potentially eligible studies.

### Study Selection

Search results were organized, and duplicate studies were identified and excluded using the EndNote X9 Program [25]. Subsequently, the results were exported to the appropriate platform for systematic reviews, Rayyan QCRI [26], for screening and analysis of articles. In the eligibility screening phase, two independent researchers reviewed the titles and abstracts of the studies and selected only those that appeared to meet the eligibility criteria. Then, the full texts of potentially eligible records were retrieved to confirm inclusion. Discrepancies were resolved by consensus or through the intermediation of a third investigator.

### Data Extraction and Risk of Bias Assessment

The characteristics of the studies and their main outcomes were extracted and tabulated according to the article name, journal/stratum name, author(s), year of publication, country of study, objectives, type of CMF, quantity of CMF samples, method of analysis, and essential and PTE concentrations. When a study had insufficient data, the authors were contacted by email at least two times.

Prior to the quantitative synthesis, extracted concentration data were checked for analytical comparability and consistency across studies. Studies reporting concentration values exceeding three standard deviations from the pooled distribution of study means were considered extreme outliers and excluded from the analysis, particularly when insufficient methodological information prevented confirmation of analytical comparability.

Risk of bias was assessed using the JBI Critical Appraisal Checklist for Analytical Cross-Sectional Studies [27]. The checklist evaluates key methodological domains, including clarity of inclusion criteria, description of the study setting and sample characteristics, validity and reliability of exposure measurement methods, identification of potential confounding factors, strategies to address confounding, and the appropriateness of statistical analyses. Each included study was independently evaluated according to these criteria. For each item, studies were classified as “Yes,” “No,” or “Unclear,” following the recommendations of the Joanna Briggs Institute.

### Meta-analysis

A narrative description of the results was performed, describing the main characteristics of the included studies. The results were evaluated according to subgroups of CMF. Data were extracted using concentration statistics (mean and standard deviation or standard error).

The meta-analysis was performed based on means and respective standard error of the PTE concentrations for the calculation of the weighted mean difference (WMD). The standard error, when not provided by the authors, was calculated using Eq. 1:

1 $$\:SE=\frac{SD}{\sqrt{N}}$$

Where SE is the standard error, SD is the standard deviation, and N is the sample size.

No imputation measures for missing data were applied. The heterogeneity of the studies used in the review was assessed by performing a Chi^2^ test (*p* < 0.1) and was considered statistically significant because of this test’s low statistical power. An I-square test (I^2^) was also adopted to test for inconsistencies across studies. Substantial heterogeneity was therefore represented when the I^2^ value exceeded 50% and visual inspection of the forest plot supported these results.

The random effects meta-analysis model using Restricted Maximum Likelihood - REML method to estimate between-study variance τ^2 was applied to the elements investigated in this review to obtain pooled effect size (weighted mean difference - WMD) in each meta analysis In this type of meta-analysis, the pooled effect size represents the levels of elements measured weighted by the number of samples analyzed in each study. Studies were stratified for subgroup analysis and identification of the possible source of heterogeneity: type of CMF (IF, FFI, and PYC) and WHO regions (AFR: African Region; AMR: Region of the Americas; EMR: Eastern Mediterranean Region; EUR: European Region; WPR: Western Pacific Region).

#### Limits of Essential and Potentially Toxic Elements in Light of Legislation

The concentrations of each element (expressed in mg kg^− 1^), obtained from meta-analysis data, were estimated as mg of element/100 kcal. For this, the average of powder per 100 kcal for each group of CMF was used as a reference (IF: 20.28 g/100 kcal; FFI: 20.78 g/100 kcal; PYC: 21.26 g/100 kcal). Then, the concentration of each element was compared with the composition requirements available in international standards.

#### Elemental Daily Intake Estimated from the Consumption of CMF

The daily intake of minerals depends on both the concentration of the elements in the food matrix and daily food consumption. The estimated daily intake (EDI) of the elements was calculated for all formula categories (IF, FFI, and PYC). We used the following equation to estimate whether the essential and trace elements intakes of IF and FFI infant formulas are adequate for infants from 0 to 6 months, 7 to 12 months old, and PYC for children 1 to 3 years old, respectively, according to Eq. 2.

2 $$\:\mathrm{E}\mathrm{D}\mathrm{I}=(\mathrm{C}\mathrm{m}\times\:\mathrm{C}\mathrm{d})/\mathrm{b}\mathrm{w}$$

Where EDI represents the estimated daily intake of each element relative to the child’s body weight (bw), expressed as mg or µg of the element per day per bw kg; *Cm* is the average concentration of each element obtained from the meta-analysis, expressed as mg g^− 1^ of formula powder; *Cd* is the recommended daily consumption amount stated on the label of each type of formula, expressed in grams of the product per day. The average intake of infant formula varies with the children age and was considered to be 780 mL/day for children from 0 to 3 months, and 1,200 mL/day from 3 to 6 months [28]. For children aged 6 to 12 months and 1 to 3 years, the volume of FFI and PYC considered was 500 mL/day, according to the literature [29–32].

Dietary adequacy was determined by comparing the EDI with the adequate intake (AI) for 0–6-month-old infants. Tolerable upper intake level (UL) was calculated only for Fe, Zn, and Se, since an established UL value for the other minerals has still not been determined (Table S4) [33, 34]. The ratio between ingested value and Tolerable Upper Intake Level (UL) was calculated. If the ratio is > 1, the child is ingesting more than the maximum limit; if it is < 1, there is no risk considering each element individually.

We compared the EDI with the Dietary Reference Intake (DRI) values proposed by the Institute of Medicine [15, 16] for trace elements (Cr, Cu, Fe, Mn, Se, and Zn) for children aged 0 to 3 years.

It should be noted that after 6 months of age, formula alone does not meet the full energy requirements of infants, as foods are introduced. Moreover, these estimates do not account for the fact that powdered formulas are reconstituted with drinking water, which also contains minerals.

### Non-carcinogenic Risk Assessment

The target hazard quotient (THQ) was developed by the Environmental Protection Agency in the US to estimate the potential human health risks (non-carcinogenic) associated with long-term chemical exposure [35] The non-carcinogenic risk of PTE intake through consumption of the dairy products was estimated according to Eq. 3:

3 $$\:\mathrm{T}\mathrm{H}\mathrm{Q}=\mathrm{E}\mathrm{D}\mathrm{I}/\mathrm{R}\mathrm{f}\mathrm{D}\mathrm{o}$$

Where:

EDI is the Estimated Daily Intake (Eq. 2), expressed as the amount of the PTE ingested daily relative to the child’s body weight (in mg per day per bw kg). The intake rate of dairy products (in kg day^− 1^) refers to an average mass needed to prepare each dairy product following label recommendations (0–6 months: 125.1 g day^− 1^, 0–12 months: 137 g day^− 1,^ and 1–3 years: 75 g day^− 1^). The body weight is: 0–6 months: 6.1 kg, 6–12 months: 9.1 kg, and 1–3 years: 12.1 kg [36].

RfDo (mg bw kg^− 1^ day^− 1^) is the oral reference dose, which values are: Al (1.0), Inorganic As (0.0003), Cd (0.0001), Co (0.0003), Cr (1.5), Cu (0.04), Fe (0.7), Methyl Hg (0.0001), Mn (0.14), Ni (0.011), Se (0.005), U (0.0002), and Zn (0.3) (19). The THQ for Pb and Ti was not calculated, since RfDo has not yet been stipulated for these elements by any competent national or international organization.

The THQ is intuitively associated with limiting exposure to non-essential, potentially toxic elements. However, when essential elements are added in excessive concentrations to fortified foods, they can also be associated with adverse health effects. Larger quantities of less bioavailable species of essential minerals are frequently incorporated in fortified food to compensate for the low absorption of the added species and to guarantee the required nutrient intake, since the most bioavailable ones are not always feasible to be employed. For this reason, it is crucial to also monitor the concentrations of essential elements, assessing their potential risks, as excessive levels can lead to bioaccumulation or deleterious health effects.

It is important to note that the RfDo for As is only available for inorganic forms, whose toxicity is considerably higher than organic As species. Since most of the included studies determined total As, the THQ was calculated considering total As or inorganic As, when available. Many studies assume that iAs represents approximately 10% of total arsenic, which is commonly found in fish, where organic forms predominate; however this proportion cannot be universally applied. In foods such as rice, for example, iAs may account for up to 50% of total arsenic, demonstrating that the inorganic fraction varies substantially depending on the food matrix. In the case of dairy products and infant formulas, this percentage is even more variable, depending on the raw materials used and, especially, on the quality of the water employed for reconstitution. Given the wide variability and the lack of speciation data for all evaluated products, a deliberately conservative approach was adopted in this work, assuming that 100% of total arsenic is present as inorganic arsenic. Obviously, this “worst-case scenario” overestimates the calculated risk and therefore, the reader should have in mind that the resulting THQ values represent the upper-bound estimates rather than the central risk one.

The total target hazard quotient (TTHQ) was expressed as the sum of the hazard quotients of each element [37]. For the analysis of the results, THQ and TTHQ values below 1 suggest an unlikely health risk, while values above 1 indicate a probable health risk, although not a cancer risk, with an increasing probability of THQ increase [35].

For the analysis of the type of formula, the minimum and maximum risk of THQ were also calculated from the 95% confidence interval of the meta-analysis, due to the heterogeneity of the analyses, and the result, whose minimum and maximum value were higher than the cut-off point, indicating risk, was considered significant. All statistical analyses were performed with STATA software, version 16.0 [38].

## Results and Discussion

### Selection Process

**Fig. 1:**
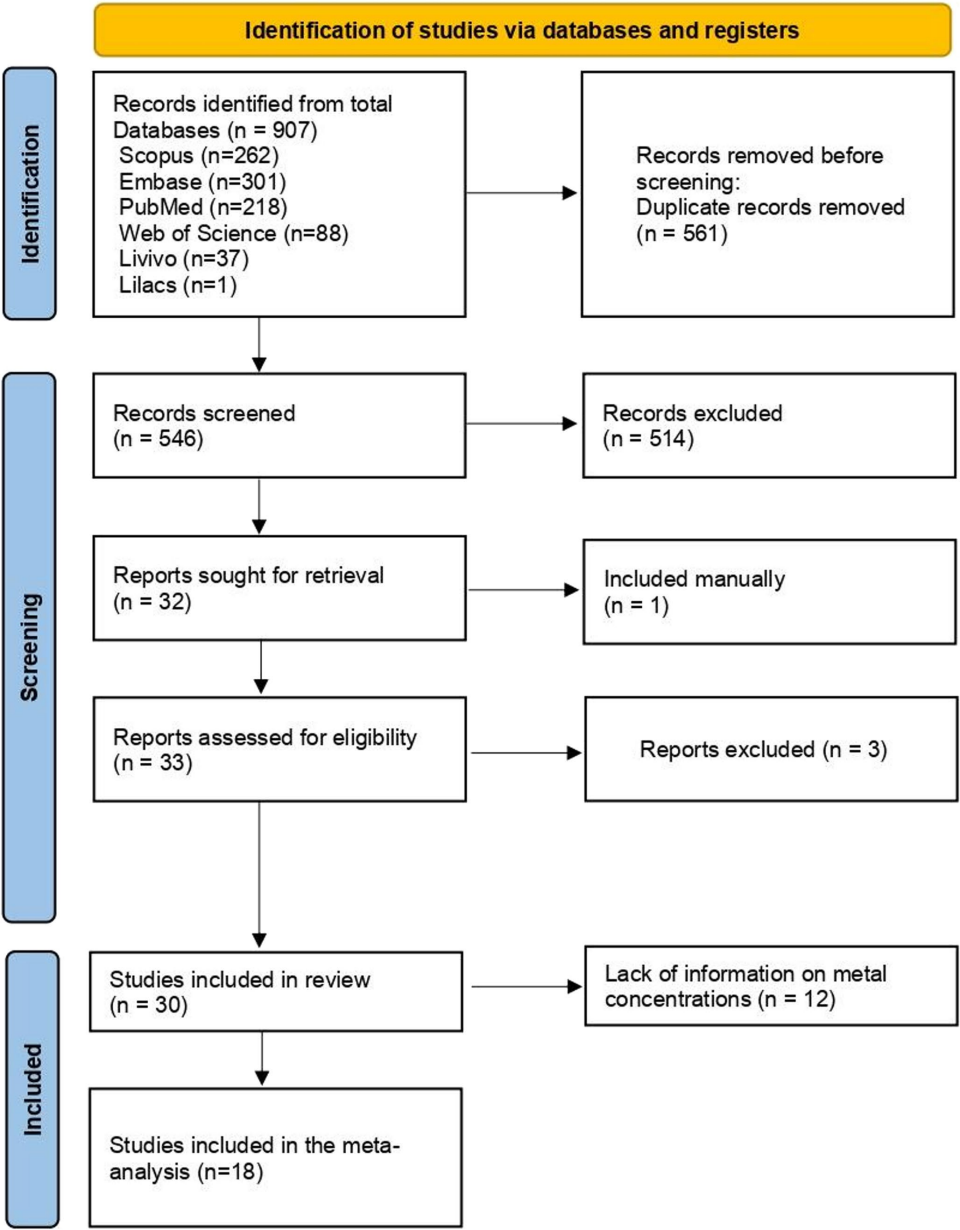
PRISMA study flow diagram for search up to February 20, 2024

The literature search identified 907 records through database searching and one additional record through citation searching. After removal of 361 duplicates, 546 records remained for title and abstract screening, of which 514 were excluded. A total of 33 reports were assessed for full-text eligibility (Table S2), and three were excluded (two validation studies and one due to non-eligible food matrix). Ultimately, 30 studies were included in the systematic review (Fig. 1). Of these, 18 studies provided sufficient data for inclusion in the meta-analysis.

### Characteristics of Studies

The characteristics of the studies included in this systematic review are presented in Table 1. Most of the retrieved studies were conducted in Europe, Asia, or the Americas (90%; *n* = 27), whereas 10% were conducted in Africa (*n* = 3). All studies were published in English.

**Table 1:** Main characteristics of the studies included in the systematic review (*n* = 30)

Author Year Country	Objective of the study	Type of CMF	Number of brands	Number of samples	Essential and PTE	Method of determination	CoI	Funding
Melo et al., 2024 Brazil [39]	To evaluate the quality of minerals-fortified milk anf young child commercialized in Brazil for young children (1–3 years), concerning the concentrations of potentially toxic elements, and to estimate the possible health risks evaluated by target hazard quotient and the target cancer risk	PYC	6	36	Al, As, Cd, Ni, Pb, Sn, Ti, U	ICP-MS	No	Public
Souza et al., 2023Brazil [40]	Evaluation of the content of essential elements and organic and inorganic pollutants in infant formulas marketed in Brazil and health risk assessment of selected contaminants.	FFI	NA	40	Pb, Cd, As, Ni e Al	ICP-MS	No	Public
Alharbi et al., 2023 Saudi Arabia [41]	Evaluation of dietary exposure and chronic non-cancer risks associated with the consumption of these products in the 0–12-month age group.	IF and FFI	NA	61	As, Cd, Pb	ICP-MS	No	Public
Almeida et al. 2022 Brazil [42]	To evaluate the content of essential elements in infant formulas for babies, assessing compliance with levels proposed by Codex Alimentarius, Brazilian standards, and label declarations.	IF and FFI	10	150	Co, Cu, Fe, Mn, Se, and Zn	ICP-MS	No	Public
de Almeida et al. 2022 Brazil [43]	To determine the level of toxic elements in infant formula brands recommended for babies and identify the potential health risk.	IF and FFI	10	150	Al, As, Cd, Methyl Hg, Pb, and U	ICP-MS	No	Public
Boon et al. 2022 Netherlands [44]	Estimate the intake of elements and assess the potential health risks.	FFI	NA	3	As, Cd, Co, Cu, Fe, Methyl Hg, Mn, Ni, Pb, Ti, and Zn	ICP-MS	No	Public
Sant’Ana et al. 2021 Brazil [23]	To evaluate the levels of heavy metals and their antagonists in dairy products.	PYC	5	10	Co, Cu, Fe, Methyl Hg, Ni, Pb, Se, and Zn	ICP OES	No	Public
Mohamed et al. 2021 Egypt [45]	Determine the elemental content of infant formula.	IF, FFI, and PYC	NA	30	Al, Co, Cu, Fe, Mn, Se, and Zn	INAA	NA	NA
Dehcheshmeh et al. 2021 Iran [46]	Evaluate heavy metals in infant formula.	IF	NA	80	Cd and Pb	GF-AAS	No	Public
Astolfi et al. 2021 Italy [17]	Determine elements in all infant formula powders.	IF	11	22	Co, Cr, Cu, Fe, Mn, Ni, Se, Ti, U, and Zn	ICP-MS	No	Public
Tahboub et al. 2021 Jordania [47]	Determine the levels of elements in infant formula.	IF	NA	22	Al, As, Cd, Co, Cr, Cu, Fe, Mn, Ni, Pb, Se, U, and Zn	ICP-MS	No	Public
Saeed et al. 2021 Pakistan, Gambia, and Australia [48]	Explore the nutritional composition as well as safety evaluation of infant formula.	IF	10	20	Al, Cd, Cu, Fe, Pb, and Zn	FAAS	NA	NA
Dobrzyńska et al. 2021 Poland [28]	Analyze the concentration of Cu and Zn in infant formulas and evaluate the Cu/Zn ratio.	IF and FFI	59	67	Cu and Zn	AAS	No	Public
Maruszewska et al. 2021 Poland [49]	Analyze the composition of essential elements as well as toxic elements in infant formulas.	IF and FFI	NA	12	Cu, Fe, Mn, Ni, Pb, and Zn	ICP OES	No	Public
Mandiá et al. 2021 Spain [50]	Quantify the concentrations of minerals and trace elements in infant formula.	IF and FFI	NA	23	Al, As, Cd, Co, Cr, Cu, Fe, Methyl Hg, Mn, Ni, Pb, Se, Ti, U, and Zn	ICP-MS/ICP OES	No	No
Marquès et al. 2021 Spain [51]	Evaluate the concentrations of essential and non-essential elements in infant formulas.	FFI	NA	14	Co, Methyl Hg, Mn, Ni, Pb, and U	ICP-MS	No	Public
Başaran, B. 2021 Turkey [52]	To detect the levels of toxic elements in infant formulas, as well as assess consumer exposure.	IF, FFI, and PYC	18	36	As, Cd, Methyl Hg, and Pb	ICP-MS	No	No
Scher et al. 2021 United States [53]	Characterize manganese concentrations in infant formulas and integrate information from these sources into a health risk assessment.	IF	22	44	Mn	ICP-MS	No	Public
Fioravanti et al. 2020 Brazil [54]	Determining the total element contents of infant formulas intended for babies.	IF and FFI	8	24	Cu, Fe, Mn, and Zn	ICP OES	NA	Public
Ibrahim et al. 2020 Egypt [55]	Define the potential health risks of toxic elements in infant formula and compare it to the allowable limits available in Egyptian standards and various international standards.	IF	NA	30	Al, As, Cd, Methyl Hg, and Pb	ICP-MS	NA	NA
Elsheikh et al. 2020 Saudi Arabia [56]	To determine the content of essential and toxic elements present in infant formulas and infant foods of different brands.	FFI and PYC	2	NA	Al, As, Cd, Mn, Ni, and Pb	ICP OES	No	Public
Chekri et al. 2019 France [20]	Determine the contamination levels of various trace elements in infant foods.	IF, FFI and PYC	NA	71	Al, As, Cd, Co, Cr, and Ni	ICP-MS	No	Public
Vella C. & Attard E. 2019 Malta [57]	Evaluate mineral content, toxic metals in infant formulas.	IF and FFI	6	18	Cr, Cu, Fe, Methyl Hg, Mn, Ni, and Zn	MP-AES	No	No
Igweze et al. 2019 Nigeria and Italy [58]	To determine the levels of essential elements in infant formulas to verify their suitability.	IF and FFI	7	21	Co, Cr, Fe, Mn, and Zn	AAS	No	No
Frisbie et al. 2019 United States and France [59]	Measure Mn concentrations in nutritional drinks for young children or follow-on formulas, which are not regulated.	IF, FFI and PYC	NA	29	Mn	PIXE/RBS	No	No
Da Silva, et al. 2018 Brazil [60]	Evaluate the concentration of essential elements in infant formulas.	IF and FFI	3	9	Cu, Fe, Mn, and Zn	ICP OES	NA	Public
Eticha et al. 2018 Ethiopia [61]	To determine the concentration of heavy metals in infant formulas, and their associated health risks to babies through consumption of these products.	FFI	5	10	​​ Cd, Pb, and Zn	FAAS	No	NA
Jallad K.N. 2018 Kuwait [21]	Determine the concentration of arsenic in various infant formulas and baby foods.	IF, FFI and PYC	10	NA	As	ICP-MS	NA	NA
Martínez et al. 2018 Spain [22]	Analyze the presence of a broad spectrum of components in infant formula samples.	IF, FFI and PYC	NA	50	Al, As, Cd, Co, Cr, Cu, Fe, Methyl Hg, Mn Ni, Pb, Se, and Zn	ICP-MS/ICP OES	NA	Public and private
Ünüvar, et al. 2017 Turkey [62]	Investigate the content of heavy metals and essential elements in different infant formulas.	IF and FFI	5	20	Al, Fe, Pb, and Zn	FAAS/GF-AAS	NA	NA

CMF: commercial milk formula. Type of CMF (IF: infant formula (*n* = 23); FFI: follow-up formula for older infants (*n* = 22); PYC: product for young children (*n* = 9)); PTE: potentially toxic elements; Methods of analysis (AAS: Atomic Absorption Spectrometry; CVAF: Cold vapor generation atomic fluorescence; FAAS: Flame Atomic Absorption Spectrometry; GF-AAS: Graphite furnace atomic absorption spectrometry; INAA: Instrumental neutron activation analysis; MP-AES: Microwave Plasma Atomic Emission Spectrometer; NAA: Neutron activation analysis; PIXE: Particle-induced X-ray emission spectrometry; RBS: Rutherford backscatter spectrometry; RNAA: Radiochemical neutron activation analysis); CoI: conflict of interest; NA: not available

Of the included studies, the number that assessed essential and PTE in CMF for infant feeding was as follows: infant formulas (*n* = 23), follow-on formulas for older infants (*n* = 22), and products for young children (*n* = 9) (Table 2).

**Table 2:** Meta-analysis of essential and potentially toxic elements concentrations (ug kg^− 1^) in different types of commercial milk formula

Elements	Product based on milk	*N* of samples in studies	WMD (95% CI)	Heterogeneity	
				*p* value	I^2^ (%)
Al	IF	10	946.75 (169.35-1724.14)	< 0.001	99.96
	FFI	7	740.02 (349.23-1130.82)	< 0.001	99.64
	PYC	9	2052.61 (173.67-3931.52)	< 0.001	99.98
_i_As	IF	10	12.2 (5.66–18.74)	< 0.001	99.94
	FFI	12	12.59 (6.86–18.32)	< 0.001	99.72
	PYC	11	23.99 (10.82–37.17)	< 0.001	99.92
Cd	IF	9	4.13 (2.86–5.41)	< 0.001	97.49
	FFI	6	3.36 (1.06–5.65)	< 0.001	99.83
	PYC	8	6.44 (3.64–9.25)	< 0.001	98.46
Co	IF	19	11.55 (7.26–15.84)	< 0.001	99.96
	FFI	6	7.91 (4.04–11.78)	< 0.001	84.52
	PYC	1	0.90 (0.80-1.00)	-	-
Cr	IF	10	250.37 (148.97-351.77)	< 0.001	99.96
	FFI	9	261.89 (162.26-361.52)	< 0.001	99.92
	PYC	1	27.70 (16.39–39.01)	-	-
Cu	IF	25	1882.7 (1366.62-2398.77)	< 0.001	100.00
	FFI	12	1947.63 (1166.61-2728.65)	< 0.001	99.99
Fe*	IF	26	34.28 (26.45–42.1)	< 0.001	99.99
	FFI	13	45.07 (27.36–62.78)	< 0.001	100.00
	PYC	1	8.89 (6.58–11.21)	-	-
methyl Hg	IF	3	10.26 (0-29.52)	< 0.001	99.99
	FFI	2	0.39 (0-1.06)	< 0.001	95.19
	PYC	1	0.14 (0-0.03)	-	-
Mn	IF	15	1566.66 (774.59-2358.72)	< 0.001	100.00
	FFI	13	1491.78 (752.54-2231.03)	< 0.001	100.00
	PYC	2	649.86 (0-1649.44)	< 0.001	99.87
Ni	IF	4	131.14 (0-336.7)	< 0.001	95.40
	FFI	4	406.35 (0-864.59)	< 0.001	100
	PYC	7	1358.2 (761.2-1955.21)	< 0.001	99.80
Pb	IF	8	42.98 (7.6-78.36)	< 0.001	99.99
	FFI	11	45.5 (16.33–74.66)	< 0.001	99.98
	PYC	7	16.9 (0-37.48)	< 0.001	99.92
Se	IF	19	180.64 (132.21-229.06)	< 0.001	99.91
	FFI	7	336.88 (107.49-566.27)	< 0.001	99.95
	PYC	1	20.00 (11.65–28.35)	-	-
U	IF	16	3.19 (1.61–4.77)	< 0.001	100.00
	FFI	6	6.12 (1.72–10.51)	< 0.001	99.62
	PYC	6	6.65 (4.19–9.11)	< 0.001	92.47
Zn*	IF	15	26.84 (18.25–35.43)	< 0.001	99.99
	FFI	18	31.31 (22.54–40.08)	< 0.001	100.00

*concentration in mg kg^− 1^; WMD: weighted mean difference; CI: confidence interval; IF: infant formula; FFI: follow-up for older infants; PYC: product for young children.Values below the LOD were assigned as zero in the meta-analysis

Concerning the analytical techniques developed to measure the PTE in these formulations, the three main ones identified were: ICP-MS (47%) > AAS (20%) > ICP OES (17%) (Table 1). These results can be attributed to the high sensitivity, low limits of detection (LOD), and multi-elemental analysis of these techniques, as well as they can also attend to the precepts of green chemistry [63].

The meta-analysis could only be carried out on eighteen studies. Nine studies [28, 40, 41, 44, 45, 49, 51, 58, 59] did not present the concentrations in means and standard deviation or standard error. In addition, three studies [23, 48, 55] reported concentration values exceeding three standard deviations from the pooled distribution of study means and lacked sufficient methodological detail to ensure analytical comparability. Despite two attempts to contact the authors by e-mail, information on metal concentrations was not returned.

The ranking order of the number of studies included in the meta-analysis according to continent was as follows: Asia, including Turkey (2) [52, 62], Iran [46], Saudi Arabia [56], Jordan [47], Kuwait [21] (6 studies, 33%) ~ America, includin Brazil (5) [39, 42, 43, 54, 60], United States (60) (6 studies, 33%) > Europe, including Spain (2) [22, 53] Italy [17], France [20], Malta [50] (5 studies, 28%) > Africa, including only Ethiopia [57] (6%). One stuy received private funding to conduct its reearch but declared no conflict of interest.

### Risk of Bias Assessment

Overall, most studies demonstrated high methodological quality according to the JBI appraisal [27]. Specifically, 80% (24/30) of the included studies were classified as high quality, 16.7% (5/30) as moderate quality, and only 3.3% (1/30) as low quality in the overall assessment.

Across individual domains, all studies adequately described the study subjects and setting (100%), while high levels of adequacy were also observed for the clarity of inclusion criteria (90%) and the validity and reliability of exposure measurement (83.3%). The identification of potential confounding factors represented the most critical domain, with only 30% of studies rated as “Yes” and the majority classified as “Unclear” (66.7%). Similarly, strategies to address confounding factors showed lower reporting clarity, with 63.3% rated as “Yes”. In contrast, the use of objective measurement criteria, the validity of outcome measurement, and the appropriateness of statistical analysis showed generally satisfactory performance, with 80%, 80%, and 76.7% of studies rated as “Yes,” respectively.

Overall, the included studies presented predominantly low risk of bias, with limitations mainly related to the identification and management of potential confounding factors. Detailed results of the risk of bias assessment are presented in Figure S1.

### The Concentration of Essential and PTE in Dairy Products According to Meta-analysis and Assessment Considering the Precepts of International Legislation

To our knowledge, this is the first systematic review study with meta-analysis that investigated a large number of elements (essential and potentially toxic) in different types of CMF intended for children between 0 and 3 years old. Although the proven benefits are much lower than human milk, more than half of infants, babies, and young children around the world use CMF as the basis of their diet [5, 61], confirming the importance of research about PTE in these products and their impact on children’s health. In addition, sales of these products represent a growing and highly lucrative market, which has already reached around 55 billion dollars a year since 2019 [4].

According to the results obtained in Table 2, the rank order of elements concentration in IF was Fe > Zn > Cu > Mn > Al > Cr > Se > Ni > Pb > _i_As > Co > methyl Hg > Cd > U, while in FFI was Fe > Zn > Cu > Mn > Al > Ni > Se > Cr > Pb > _i_As > Co > U > Cd > methyl Hg. In the PYC, the rank order was Fe > Al > Ni > Mn > Cr > _i_As > Se > Pb > U > Cd > Co > methyl Hg.

In this study, the concentrations (mg of element per 100 kcal) of some essential minerals (Fe, Mn, Se, and Zn) and PTE (Cd and Pb) reported in the IF and FFI studies (Table S5) were within the maximum and minimum limits established by legislation [61, 64–66].

Copper, on the other hand, although was within the minimum limit for IF and FFI (38.18 µg/100 kcal) recommended by Codex Alimentarius (35 µg/100 kcal) [2] it was lower than two other regulatory bodies that recommend a minimum of 60 µg/100 kcal [65, 66]. Copper is an essential element to many important biological processes in humans, mainly in children, acting as a cofactor of several enzymes (ceruloplasmin, cytochrome oxidase, catechol oxidase), including electron transport, in iron metabolism, in the scavenging of free radicals, as well as in many neurological functions [67].

Concerning PYC, there is very limited data on the minimum and maximum levels of elements stipulated by international government bodies. Codex Alimentarius established 1–3 mg/100 kcal for Fe, but concentrations below the recommended level were observed [3].

Still about PTE, the average concentrations of inorganic As identified in this systematic review for IF (12.2 µg kg^− 1^) and FFI (12.59 µg kg^− 1^) were below the limit set by the Commission Regulation (EU) (0.02 mg kg^− 1^, i.e., 20 µg kg^− 1^), while only PYC (24 µg kg^− 1^) were slightly above, which may represent a health risk for children, indicating the urgent need to monitor this product on the market [68].

Arsenic is among the most toxic elements in the world, especially the inorganic compounds (_i_As), as widely found in a variety of environments, and they have been classified as ‘carcinogenic to humans’ by the International Agency for Research on Cancer [69–71]. Children are exposed to arsenic in many of the same ways as adults. Drinking infant formula made with arsenic-contaminated water can be a significant source of exposure. The main symptoms of arsenic poisoning include irritation of the stomach and intestines, blood vessels damage, skin changes, and reduced nerve function. There is also some evidence to suggest that long-term exposure to inorganic arsenic in children can result in lower IQ scores. The Agency for Toxic Substances and Disease Registry [72] does not clarify whether the absorption of inorganic arsenic from the gut in children is different from that in adults, since in the reported results, the participants are not assessed by age group, but are divided by sex [73]. Despite this, most studies have determined total As, but the inorganic form of As is highly related to toxicity compared to the other forms [74]. So, the values found slightly above the maximum limit indicate the need to determine the species of As found in the samples, which may or may not pose a significant risk, depending on the species. In addition, it is important to note that organic forms and macromolecules of arsenic may be less toxic than inorganic arsenic (_i_As), but they are not free from causing toxicity in cases of high exposure [75]. The remaining PTE, such as Al, Co, Cr, methyl Hg, Ni, and U, are frequently cited in the ATSDR list of hazardous substances [76]. For instance, the safe intake of Al and Ni in food has also been discussed in other EFSA documents [77, 78], emphasizing the importance of establishing limit levels in children’s products. A detailed discussion of the health risk assessment will be covered in Elemental daily intake estimated from the consumption of CMF. 

In relation to the essential elements investigated in this review (Fe, Zn, Cu, Se, Cr, and Mn), when present within the values of the RDI standards, they help stabilize cell structures and biochemical and biological activities. On the other hand, low concentrations in the body can cause diseases and, above certain limits, can be toxic and cause deleterious effects on human health, especially in children, reaffirming once again the constant importance of monitoring the nutritional composition of these products on the market [79, 80].

### Elemental Daily Intake Estimated from the Consumption of CMF

Essential and trace element daily intakes, based on the Institute of Medicine [34] reference values for children aged 0–6 months (IF), 7–12 months (FFI), and > 12 months (PYC), are presented in Supplementary Table 4. The estimated average intakes of Cr, Cu, Mn, Zn, and Se in the IF group, calculated through meta-analysis, met the recommended AI values for infants aged 0–6 months. In the 0–3 months subgroup, Mn, Fe, and Cr intakes were approximately 55-, 13-, and 132-fold higher than the AI, respectively. In the 3–6 months subgroup, these values exceeded the AI by 85-, 20-, and 200-fold, respectively. Zn intake also surpassed the UL by 1.1-fold in this group.

Mn intake above nutritional recommendations was consistently observed in IF products, corroborating previous reports [81–83]. According to EFSA, 3 µg/day of Mn is adequate during the first six months of life, increasing to 20–500 µg/day in the second year [78]. Although Mn is essential for enzymatic activity and macronutrient metabolism, elevated intake may exert neurotoxic effects [84, 85]. Liu [86] demonstrated that elevated Mn exposure adversely affects cognitive and motor development in children < 6 years consuming IF, FFI, and PYC [87] Exposure to high concentrations of Mn in drinking water has been associated with adverse neurological outcomes in school-aged children [59]. Infant formulas generally contain substantially higher Mn concentrations than breast milk, which may increase exposure to this element in formula-fed infants [59, 88]. Although regulatory agencies in the country have established tolerable intake levels for this element, there is still limited evidence regarding the safety of these levels for the neurological development of infants. In this context, these findings highlight the importance of monitoring Mn levels in powdered products, particularly considering regional variations in the mineral content of the water used for reconstitution.

In the FFI group, formula intake met the daily requirements only for Cr and Se, in line with previous findings [30]. Piccinelli [32] reported that infant formula provides ~ 45% of energy requirements at 7 months and 25% at 10 months [32]. For IF (0–3 and 3–6 months) and FFI, Se intake exceeded the AI by 1.27-, 1.96-, and 1.17-fold, respectively - an important finding given the risk of Se deficiency in infants and preschool children [82]. Iron intake was also markedly above the AI in IF (13.45- and 20.69-fold for 0–3 and 3–6 months, respectively), while intakes in FFI and PYC fell below the AI. However, children in these latter groups consume complementary foods that provide additional Fe.

Essential and trace elements are indispensable for physiological processes such as enzymatic reactions, bone mineralization, and cell protection, directly influencing growth and development [42, 89]. Deficient intakes or low bioavailability may impair development [90, 91]. The World Health Organization (WHO) emphasizes that daily requirements must sustain physiological function and prevent deficiency [92, 93]. To compensate for low bioavailability, manufacturers often add minerals at concentrations exceeding those of breast milk, which may affect the accuracy of label declarations [2]. In this study, Mn intakes in IF were 55.13- and 85.12-fold above AI for 0–3 and 3–6 months, respectively. This exposure may be further exacerbated by environmental factors and the mineral composition of water used in reconstitution [87].

Several minerals can cause adverse effects when consumed chronically above the UL. In the present study, only Zn exceeded the UL in IF, consistent with prior evidence [42]. Prolonged Zn intake above the UL may impair Cu absorption, induce anemia, and compromise immune function [94, 95]. Importantly, the absence of established UL for some elements, such as Cr and Mn, does not preclude the harmful effects of excessive intake [42, 59, 88].

Regarding Fe, cow’s milk contains insufficient concentrations to meet infant requirements, reinforcing the recommendation against its use in children < 12 months [96, 97]. An infant under 6 months would require an unrealistically high volume of cow’s milk to reach the recommended Fe intake. By contrast, many formulas are fortified with Fe (8–14 mg/L), compared with only ~ 0.3 mg/L in breast milk [98]. This higher amount is to compensate for the different bioavailability of the Fe species supplemented in the formulas compared to the species in the breastmilk [99, 100].

### PTE Level in Dairy Products According to the World Health Organization Classification of Regions

Based on the results in Table 3, the PTE concentrations in the infant formulas were different between the continents studied. The highest concentrations of the elements Al (4.6 mg kg^− 1^), Cd (6.10 µg kg^− 1^), Co (55.40 µg kg^− 1^), Cr (588 µg kg^− 1^), Fe (65.20 mg kg^− 1^), Ni (344 µg kg^− 1^), and Zn (32.90 mg kg^− 1^) were related to the Eastern Mediterranean Region, while the highest concentrations of Cu (2.67 mg kg^− 1^), Mn (1.8 mg kg^− 1^), Se (344.52 µg kg^− 1^) and U (8.36 µg kg^− 1^) belonged to Region of the Americas and the highest concentrations of Pb (53.68 µg kg^− 1^) and _i_As (9.30 µg kg^− 1^) belonged to the European Region.

**Table 3:** Meta-analysis of essential and potentially toxic elements concentrations (ug kg^− 1^) in different types of commercial milk formula, according to WHO regions

Elements	WHO regions	*N* of samples in studies	WMD (95% CI)	Heterogeneity	
				*p* value	I^2^ (%)
Infant formula					
Al	AMR	5	570.21 (439.42–701)	< 0.001	96.28
	EUR	4	530.55 (0-1099.05)	< 0.001	99.66
	EMR	1	4600 (3889.87-5310.13)	-	-
_i_As	AMR	3	22.41 (13.43–31.39)	< 0.001	97.82
	EUR	3	9.3 (0-29.21)	< 0.001	99.99
	EMR	4	8.07 (4.75–11.39)	< 0.001	98
Cd	AMR	5	4.51 (3.98–5.03)	0.139	43.21
	EUR	2	1 (0-2.67)	0.078	67.84
	EMR	2	6.1 (3.23–8.97)	0.530	73.20
Co	AMR	5	10 (7.74–12.26)	1.00	0.00
	EUR	13	9.36 (6.98–11.74)	< 0.001	99.88
	EMR	1	55.4 (46.29–64.51)	-	-
Cr	AMR	5	246.91 (231.07-262.76)	0.002	80.89
	EUR	4	172.75 (0-359.25)	< 0.001	99.99
	EMR	1	588 (516.8-659.21)	-	-
Cu	AMR	10	2672.33 (1667.44-3677.22)	< 0.001	99.99
	EUR	14	1279.11 (914.69-1643.52)	< 0.001	99.98
	EMR	1	2420 (2225.65-2614.35)	-	-
Fe*	AMR	10	42.95 (27.57–58.33)	< 0.001	99.99
	EUR	15	26.36 (20.15–32.57)	< 0.001	99.97
	EMR	1	65.2 (58.96–71.44)	-	-
methyl Hg	EUR	3	10.26 (0-29.52)	< 0.001	99.99
Mn	AMR	11	1845.78 (832.66-2858.91)	< 0.001	100.00
	EUR	3	784.76 (0-2091.4)	< 0.001	99.96
	EMR	1	984 (804.78-1163.22)	-	-
Ni	EUR	3	18.05 (0-49.94)	0.141	0.00
	EMR	1	344 (169.54-518.46)	-	-
Pb	AMR	2	23.93 (4.75–43.11)	0.09	64.62
	EUR	4	53.68 (0-124.26)	0.00	99.95
	EMR	2	38.6 (0-89.88)	0.00	99.47
Se	AMR	5	344.52 (298.07-390.98)	< 0.001	83.47
	EUR	13	125.65 (97.57-153.74)	< 0.001	99.74
	EMR	1	120 (106.56-133.45)	-	-
U	AMR	3	8.36 (4.92–11.8)	< 0.001	98.90
	EUR	12	1.91 (0.86–2.97)	< 0.001	99.99
	EMR	1	3.02 (2.63–3.41)	-	-
Zn*	AMR	10	30.33 (19.2-41.48)	< 0.001	99.99
	EUR	4	16.68 (3.33–30.03)	< 0.001	99.87
	EMR	1	32.9 (29.35–36.45)	-	-
Follow-up for older infants					
Al	AMR	4	838.25 (516.68-1159.82)	< 0.001	98.46
	EUR	3	615.47 (0-1520.29)	< 0.001	99.77
_i_As	AMR	5	21.25 (13.7-28.79)	< 0.001	95.92
	EUR	3	1.67 (1.48–1.87)	0.23	0.00
	EMR	4	7.17 (6.52–7.86)	0.13	44.71
Cd	AMR	4	4.92 (3.07–6.78)	< 0.001	93.66
	EUR	2	0.253 (0-0.6)	< 0.001	95.02
Co	AMR	5	10 (7.74–12.26)	1.00	0.00
	EUR	1	0.25 (0.19–0.31)	-	-
Cr	AMR	5	340.18 (295.69-384.68)	< 0.001	92.81
	EUR	4	165.08 (0-350.08)	< 0.001	99.98
Cu	AMR	9	2127.23 (1262-2992.46)	< 0.001	99.99
	EUR	3	1409.37 (0-3331.18)	< 0.001	99.95
Fe*	AMR	9	52.94 (32.08–73.8)	< 0.001	100.00
	EUR	4	27.33 (0-57.07)	< 0.001	99.98
methyl Hg	EUR	2	0.39 (0-1.06)	< 0.001	95.19
Mn	AMR	9	1853.3 (935.79-2770.81)	< 0.001	100.00
	EUR	3	750.83 (0-2021.91)	< 0.001	99.97
	EMR	1	459 (413.74-504.26)	-	-
Ni	EUR	4	406.35 (0-864.59)	< 0.001	100.00
Pb	AMR	2	13.98 (3.82–24.14)	0.159	49.54
	EUR	4	60.92 (0-135.97)	< 0.001	99.91
	EMR	1	57 (53.61–60.39)	-	-
	AFR	4	35.43 (0.04–70.81)	0.459	10.54
Se	AMR	5	462.5 (223.98-701.02)	< 0.001	99.32
	EUR	2	22.51 (10.45–34.56)	0.03	78.64
U	AMR	5	7.22 (2.49–11.95)	< 0.001	99.37
	EUR	1	0.7 (0.35–1.05)	-	-
Zn*	AMR	9	28.59 (16.59–40.59)	< 0.001	100.00
	EUR	4	19.74 (3.84–35.64)	< 0.001	99.91
	AFR	5	45.43 (31.35–59.5)	< 0.001	99.89
Product for young children					
Al	AMR	6	1522.47 (184.7-2860.23)	< 0.001	99.79
	EUR	2	256.07 (77.38-434.76)	0.07	69.94
	EMR	1	8690 (8169.47-9210.53)	-	-
_i_As	AMR	6	40.03 (24.79–54.27)	< 0.001	98.92
	EUR	2	1.614 (0.5–2.73)	0.041	75.98
	EMR	3	7.4 (5.55–9.24)	< 0.001	89.31
Cd	AMR	6	8.24 (6.12–10.37)	< 0.001	95.54
	EUR	2	1.01 (0.427–1.59)	0.31	3.42
Co	EUR	5	0.9 (0.802–0.998)	< 0.001	-
Cr	EUR	1	27.7 (16.39–39.01)	-	-
Fe*	EUR	1	8.89 (6.58–11.21)	-	-
methyl Hg	EUR	1	0.14 (0-0.3)	-	-
Mn	EUR	1	140 (94.04-185.96)	-	-
	EMR	1	1160 (1103.42-1216.58)	-	-
Ni	AMR	6	1490.97 (856.54-2125.39)	< 0.001	99.83
	EUR	1	550 (341.07-758.93)	-	-
Pb	AMR	5	5.36 (2.53–8.2)	< 0.001	94.05
	EUR	1	6.96 (5.33–8.59)	-	-
	EMR	1	79 (75.49–82.51)	-	-
Se	EUR	1	20 (11.65–28.35)	-	-
U	AMR	6	6.65 (4.2–9.11)	< 0.001	92.47

*concentration in mg kg^− 1^; WMD: weighted mean difference; CI: confidence interval; WHO: World Health Organization; WHO Regions (AFR: African Region; AMR: Region of the Americas; EMR: Eastern Mediterranean Region; EUR: European Region; WPR: Western Pacific Region).Values below the LOD were assigned as zero in the meta-analysis

The highest PTE concentrations in the follow-up for older infants formulas (Table 3) were Al (838.2 µg kg^− 1^), _i_As (21.2 µg kg^− 1^), Cd (4.9 µg kg^− 1^), Co (10 µg kg^− 1^), Cr (340.2 µg kg^− 1^), Cu (2.13 mg kg^− 1^), Fe (52.9 mg kg^− 1^), Mn (1.9 mg kg^− 1^), Se (462.5 µg kg^− 1^), and U (7.2 µg kg^− 1^) in Region of the Americas, while the highest concentrations of methyl Hg (0.39 µg kg^− 1^), Ni (406.3 µg kg^− 1^) and Pb (60.9 µg kg^− 1^) were related to the European Region, and the higher concentration of Zn (45.4 mg kg^− 1^) in the African Region.

Regarding the product for young children, the highest concentrations of PTE were Al (1522.5 µg kg^− 1^) and As (7.4 µg kg^− 1^) in the Region of the Americas, while the highest concentrations of Ni (550 µg kg^− 1^), Cr (27.7 µg kg^− 1^), Se (20 µg kg^− 1^), Fe (8.9 mg kg^− 1^), Pb (6.96 µg kg^− 1^), Co (0.90 µg kg^− 1^), and methyl Hg (0.14 µg kg^− 1^) were in the European Region. The highest concentration of Mn (1.2 mg kg^− 1^) was observed in the Eastern Mediterranean Region. Based on the results, a wide variety of element concentrations was found according to the regions identified in this study. The differences obtained may be related to the characterization of the geographical origin, applied plant species, and water for animal consumption, weather conditions, industrial and urban activities near heavy traffic roads, factories, mines, use of chemicals such as fertilizers or pesticides, and atmospheric conditions [11].

The physicochemical properties of metals can also explain the differences found in the studies and between the products analyzed, according to the different geographical regions. Other studies have shown that the low concentrations of metals in some regions may be due to the physicochemical properties of the soil, and the climatic conditions of the region may alter the metal content. This can make these metals inaccessible to plants and, consequently, to animals [46].

Exposure to the environment or the consumption of food and water by livestock can lead to PTE contamination of primary milk, which can result in the occurrence of elemental contamination in dairy products [46]. In parallel, there are differences in regulations by geographical region when it comes to drinking water and contaminants. Regarding manganese levels, for example, in 2019, Canada implemented a standard for manganese in public drinking water based on the protection of children [101]. Nonetheless, the WHO has also proposed reducing its guidance value based on protecting formula-fed babies, the subpopulation most susceptible to exposure to this metal [102]. On the other hand, in the United States, manganese is an unregulated contaminant. It should be noted that this is a country where infant formula feeding is common, with less than 50% of babies and around 25% being exclusively breastfed for the first 3 and 6 months of life, respectively. Therefore, in this country, the population is susceptible to consuming manganese in higher doses, since more than one-third of Americans use groundwater for consumption, which is more likely to have higher concentrations of manganese than surface water [88].

### Health Risk Assessment

During the first 6 months of life, breast milk provides all the nutrients and calories needed for babies to develop and grow [11]. However, for various reasons, CMF is introduced into the baby’s diet as early as this stage as a complement to the diet or, in some cases, as a major source of food [41]. Although essential nutrients (such as arachidonic and docosahexaenoic acids) for children’s growth and development are present in CMF, these products can contain elements that, in excess, become toxic to the body, putting the health of babies and children at risk. Compared to adults, children are more vulnerable to these contaminants because their blood-brain barrier, ability to bind to plasma proteins, enzymatic clearance processes in the liver and kidneys, and immune systems are underdeveloped [11].

For the assessment of the health risk to children caused by the consumption of PTE present in CMF, the Target Hazard Quotient (THQ), well known as the non-carcinogenic risk, was estimated using the concentrations of PTE expressed in weighted mean difference from the meta-analysis results (Table 3). In addition, the minimum and maximum values of the THQ were also calculated from the 95% confidence interval of the meta-analysis. If THQ > 1, children are at health risk.

When THQ was evaluated considering the mean concentrations and by formula type (Table 4), the IF products had values for methyl Hg, Fe, and Zn greater than 1. Considering the worst scenario, i.e., the maximum concentrations, _i_As, Cd, Co, and Cu also presented a THQ value higher than 1. For FFI, Se and Zn showed THQ > 1, considering their mean concentrations. Furthermore, it is important to note that Ni, Fe, Se and Cu also presented THQ > 1, for the maximum concentrations in the FFI. The latter group (PYC) also had a THQ value for Ni higher than 1 for the maximum concentrations, but lower than 1 for the mean concentration. Thus, considering all samples around the world for the elements mentioned above, children aged 0 to 3 years may be subject to a non-carcinogenic health risk due to exposure to these elements for each category of products based on milk.

**Table 4:** Target hazard quotient (THQ) in different types of commercial milk formula for children between 0 and three years old

Elements	IF			FFI			PYC		
	Mean	Min	Max	Mean	Min	Max	Mean	Min	Max
Al	0.019	0.003	0.035	0.011	0.005	0.017	0.013	0.001	0.024
_i_As	0.834	0.387	**1.281**	0.632	0.344	0.919	0.496	0.224	0.768
Cd	0.847	0.587	**1.109**	0.506	0.16	0.851	0.399	0.226	0.573
Co	0.79	0.496	**1.083**	0.397	0.203	0.591	0.019	0.017	0.021
Cr	0.003	0.002	0.005	0.003	0.002	0.004	nd	nd	nd
Cu	0.965	0.701	**1.23**	0.733	0.439	**1.027**	nd	nd	nd
Fe	**1.004**	0.775	**1.233**	0.969	0.588	**1.35**	0.079	0.058	0.099
methyl Hg	**2.104**	nd	**6.054**	0.059	nd	0.16	0.009	nd	0.019
Mn	0.229	0.113	0.346	0.16	0.081	0.24	0.029	nd	0.073
Ni	0.244	nd	0.628	0.556	nd	**1.183**	0.765	0.429	**1.102**
Se	0.741	0.542	0.94	**1.014**	0.324	**1.705**	0.025	0.014	0.035
U	0.327	0.165	0.489	0.461	0.129	0.791	0.206	0.13	0.282
Zn	**1.835**	**1.248**	**2.422**	**1.571**	**1.131**	**2.011**	nd	nd	nd

nd: not determined. IF: infant formula; FFI: follow-up for older infants; PYC: product for young children. In bold are THQ higher than 1. THQ ≤ 1, indicates negligible risk; THQ > 1, indicates potential harmful effects on children’s health

On the other hand, with the aim to assess individual exposure to toxic elements in detail, the THQ was also calculated considering the formula category (IF, FFI, and PYC) by different countries (Table 5). For IF, THQ values (Table 5) higher than 1 were observed for _i_As in Turkey (2.9) and Brazil (1.5); Cd in Jordan (1.6) and Iran (1.0), Co in Jordan (3.8); Cu in Malta (1.7), Brazil (1.4) and Jordan (1.2); Fe in Jordan (1.9), Turkey (1.8) and Brazil (1.3); methyl Hg in Spain (3.2); Ni in Spain (1.2); Se in Brazil (1.4); Zn in Jordan (2.3), Brazil (2.1), Turkey (2.0) and Malta (1.9). These results indicate a non-carcinogenic risk in the consumption of IF, and therefore, the concentration of _i_As, Co, Cd, Cu, Fe, methyl Hg, Ni, Se, and Zn in IF products should be effectively monitored in the respective countries.

**Table 5:** Average THQ and TTHQ values in different types of commercial milk formulas, according to different countries

Country	Type of CMF	Al	_i_As	Cd	Co	Cr	Cu	Fe	methyl Hg	Mn	Ni	Se	U	Zn	TTHQ
Brazil	**IF**	0.012	**1.532**	0.925	0.684	0.003	**1.37**	**1.258**	-	0.295	-	**1.413**	0.857	**2.073**	**10.423**
	**FFI**	0.013	**1.066**	0.741	0.502	0.003	0.801	**1.139**	-	0.199	-	**1.393**	0.544	**1.435**	**8.834**
	**PYC**	0.009	0.827	0.511	-	-	-	-	-	-	0.84	-	0.206	-	**2.393**
Ethiopia	**FFI**	-	-	-	-	-	-	-	-	-	-	-	-	**2.28**	**2.28**
France	**IF**	0.004	0.11	0.08	0.062	0	-	-	-	-	0.048	-	-	-	0.305
	**FFI**	0.004	0.084	0.065	-	0	-	-	-	-	0.036	-	-	-	0.19
	**PYC**	0.001	0.044	0.044	0.019	0	-	-	-	-	-	-	-	-	0.107
Iran	**IF**	-	-	**1.019**	-	-	-	-	-	-	-	-	-	-	**1.019**
Italy	**IF**	-	-	-	0.752	-	0.635	0.82	-	-	-	0.592	0.209	-	**3.008**
Jordan	**IF**	0.094	0.321	**1.641**	**3.787**	0.008	**1.241**	**1.91**	-	0.144	0.641	0.492	0.31	**2.249**	**12.839**
Kuwait	**IF**	-	0.63	-	-	-	-	-	-	-	-	-	-	-	0.63
	**FFI**	-	0.36	-	-	-	-	-	-	-	-	-	-	-	0.36
	**PYC**	-	0.153	-	-	-	-	-	-	-	-	-	-	-	0.153
Malta	**IF**	-	-	-	-	0.004	**1.707**	0.537	-	0.312	-	-	-	**1.862**	**4.423**
	**FFI**	-	-	-	-	0.002	**1.268**	0.406	-	0.22	**1.122**	-	-	**1.656**	**4.675**
Saudi Arabia	**FFI**	-	-	-	-	-	-	-	-	0.049	-	-	-	-	0.049
	**PYC**	0.054	-	-	-	-	-	-	-	0.051	-	-	-	-	0.105
Spain	**IF**	0.006	0.034	-	0.017	0.003	0.228	0.175	**3.15**	0.018	**1.171**	0.117	0.057	0.334	**5.311**
	**FFI**	0.001	0.07	-	0.013	0.002	0.16	0.194	0.113	0.011	0.545	0.068	0.053	0.284	**1.513**
	**PYC**	0.002	-	-	-	-	-	0.079	-	0.006	0.31	0.025	-	-	0.422
Turkey	**IF**	0.027	**2.938**	0.449	0.752	-	-	**1.797**	0.027	-	-	-	-	**2.032**	**8.02**
	**FFI**	0.023	0.878	0.012	-	-	-	**1.561**	0.009	-	-	-	-	**1.738**	**4.222**
	**PYC**	-	0.02	0.081	-	-	-	-	0.009	-	-	-	-	-	0.109
USA	**IF**	-	-	-	-	-	-	-	-	0.025	-	-	-	-	0.025

CMF: commercial milk formula; IF: infant formula; FFI: follow-up for older infants; PYC: product for young children; THQ: target hazard quotient; TTHQ: total target hazard quotient. In bold THQ and TTHQ higher than 1.THQ or TTHQ ≤ 1, indicates negligible risk; THQ or TTHQ > 1, indicates potential harmful effects on children’s health

In addition, the Total Target Health Quotient (TTHQ) values were also computed (Table 5). TTHQ was obtained as the sum of each THQ for the whole PTE available. In the same way, if TTHQ > 1, infants are also at a non-carcinogenic health risk. Regarding IF products, based on TTHQ, the following countries ranked first: Jordan > Brazil > Turkey > Spain > Malta > Italy > Iran > Kuwait > France > United States. The TTHQ in Jordan (12.8), Brazil (10.4), Turkey (8.0), Spain (5.3), Malta (4.4), Italy (3.0), and Iran (1.0) indicate that IF products in these countries are not safe for infants (aged 0 to 6 months) for regular consumption. In the case of FFI products (Table 5), THQ values higher than 1 were observed for _i_As in Brazil (1.1); Cu in Malta (1.3); Fe in Turkey (1.6), Brazil (1.1); Ni in Malta (1.1); Se in Brazil (1.4); and Zn in Ethiopia (2.3), Turkey (1.7), Malta (1.7), Brazil (1.4). The elements referred to should be monitored in FFI products in the respective countries as a matter of priority. Based on TTHQ higher than 1, the following countries ranked first: Brazil (7.8) > Malta (4.7) > Turkey (4.2) > Ethiopia (2.3) > Spain (1.5). These results suggest that FFI products in these countries are not safe for regular consumption by older infants (aged 6 months to 1 year).

It is important to point out that Fe had the highest concentrations for all CMF, but only IF and FFI presented THQ higher than 1 for this element. Iron plays an essential role in various biological reactions in the human body, and particularly in infants, its deficiency significantly impacts health, such as impairing cognitive development and causing anemia [63, 98, 103]. Thus, to avoid a deficiency of this nutrient in their feeding and due to issues related to iron bioavailability, infant formulas are generally supplemented with this essential element [98]. This practice can lead to higher concentrations of these products and increase the non-carcinogenic risk.

With regard to the PYC (Table 5), there were no observations of THQ values higher than 1 for each country. Based on TTHQ, only Brazil showed values higher than 1 (TTHQ = 2.4), being _i_As (0.83), Ni (0.8), and Cd (0.5) with the highest values of THQ. However, these data should be considered with caution when evaluating TTHQ by country, since there was only one study that reported concentrations of these elements in PYC samples for Brazil. Thus, further studies are needed to more robustly analyze the non-carcinogenic risk of PYC samples, and it cannot be generalized that they are unsafe for consumption in Brazil.

Although the concentrations of elements in the CMF do not exceed the limits set by international regulatory bodies, as discussed in Risk of bias assessment, there is a risk to children’s health (THQ > 1) related to _i_As (IF: Brazil, 1.5; Turkey, 2.9 and FFI: Brazil, 1.1), Cd (IF: Iran, 1.0; Jordan, 1.6), Cu (IF: Brazil, 1.4; Jordan, 1.2; Malta, 1.7; FFI: Malta, 1.3); Fe (IF: Jordan, 1.9; Turkey, 1.8, Brazil: 1.3 and FFI: Turkey, 1.6; Brazil: 1.1); Se (Brazil: IF, 1.4; FFI: 1.4); and Zn (IF: Jordan, 2.3; Brazil, 2.1; Turkey, 2.0; Malta, 1.9 and FFI: Ethiopia, 2.3; Turkey, 1.7; Malta, 1.7; Brazil, 1.4).

In relation to the elements that do not have limits established in CMF by international regulatory bodies, we highlight those elements that have THQ > 1: Co (IF, Jordan, 3.8); methyl Hg (IF, Spain, 3.1); Ni (FFI, Malta, 1.1 and IF, Spain, 1.2). These results indicate the urgent need for these products to be investigated in their respective countries, as well as for the authorities to determine the limits for these elements in CMF, especially for Co, methyl Hg, and Ni.

Two other systematic reviews evaluated the risk of human milk consumption by children. While Ghane [11] reported that no country evaluated in their study had a TTHQ greater than 1, Neshat [63] showed that Egypt, Pakistan, Brazil, Jordan, and Turkey had a TTHQ greater than 1 [11, 63]. These differences were justified by Neshat et al. mainly due to the search strategies used, as well as differences in the calculation of THQ [63]. It should also be noted that they considered two additional elements in the TTHQ calculation (Mn and Pb). The CMF evaluated in this study also had a TTHQ above 1 for Brazil (IF, FFI, PYC), Jordan (IF), and Turkey (IF).

Rahimi et al. analyzed the combined risk of four dairy products (butter, cheese, milk, yogurt) in each country [46]. The results indicate that, except in Italy, local inhabitants are not at risk from the consumption of milk and dairy products (TTHQ < 1).

The country-level risk estimation must be carefully interpreted due to limitations in data availability. This can be explained, particularly in most developing countries, by the lack of resources required to collect and analyze data for risk assessment. Investment in food safety infrastructure remains low, and the availability of skilled and qualified personnel is limited [104]. In this case, the data absence may lead to an underestimation of the health risks in that population. On the other hand, the use of total metal concentrations and the assumption of complete bioavailability in ingestion exposure assessments introduce uncertainty and may overestimate health risks [105].

Recent studies in Latin America have also reported the occurrence of potentially toxic elements in different food matrices. Castañeda et al. identified elevated Hg and Pb concentrations and THQ values above 1 under high-exposure scenarios. Although the study focused on fruit matrices, it highlights the role of environmental factors such as soil contamination in the transfer of metals through the food chain [106]. This pathway may also influence dairy-based products, since the elemental composition of milk can be affected by soil–plant interactions and animal feeding conditions, including water.

In some South American countries, elevated cadmium (Cd) concentrations in cocoa beans have been attributed to naturally high Cd levels in the soil as well as to the use of fertilizers, while inorganic arsenic (iAs) concentrations in rice vary substantially among regions [107]. These findings reinforce the importance of monitoring PTE levels in commercial milk formulas and applying robust dietary exposure assessments, particularly for vulnerable populations, such as infants, children, and pregnant women, dietary intake levels are close to or even exceed the recommended values [108].

In general, a greater tendency towards non-carcinogenic risk was observed in the consumption of IF and FFI than in that of PYC. Nonetheless, different patterns of THQ and TTHQ were found, which may be due to different concentrations of PTE in various countries, as well as individual characteristics of geography, methods of processing food, and analytical methods. In an initial assessment, these data may demonstrate a potential risk of intoxication in babies and children by some PTE in several studies we reviewed, causing concern for the health and development of each of these children in several countries analyzed in these studies.

Recent studies on dietary exposure to potentially toxic elements highlight methodological challenges in risk estimation. In this review, the total target hazard quotient (TTHQ), calculated as the sum of individual THQ values, should be interpreted with caution. This approach may overestimate risk because the effects of simultaneous exposure to multiple PTEs are not strictly additive and antagonistic interactions may attenuate potential health impacts [109]. Conversely, limited exposure data and incomplete reporting of elemental concentrations may also lead to underestimation of risk [110].

Additional limitations include the use of aggregated country-level data, which may not reflect regional variability in dietary patterns, and assumptions regarding consumption rates due to incomplete information in the included studies. Despite these constraints, this review addresses an important scientific gap by synthesizing globally dispersed evidence on elemental concentrations in commercial milk formulas, a field in which comparable global analyses and quantitative meta-analyses of dietary risk remain scarce.

## Conclusion

The meta-analysis was carried out to evaluate the estimated concentrations of essential elements and PTE in the CMF consumed by children aged 0 to 3, and according to the WHO regions in the world. According to meta-analysis results, Al, Cu, Cr, Fe, Mn, Ni, Se, and Zn showed the highest concentrations among IF and FFI, while for the PYC, the elements Fe, Al, Ni, and Mn presented the highest levels. With regard to WHO regions, most of the elements with the highest concentrations in the IF were found in the Eastern Mediterranean Region. In the case of FFI, these were in the Region of the Americas, and the PYC in the regions of Europe and the Americas.

The health risk assessment for infants and children was evaluated using the non-carcinogenic risk based on the results of the meta-analysis. The assessment of the total non-cancer risk of essential elements and PTE in CMF revealed a considerable effect (TTHQ > 1) on children’s health, according to the type of CMF: (a) IF: Jordan > Brazil > Turkey > Spain > Malta > Italy > Iran; (b) FFI: Brazil > Malta > Turkey > Ethiopia > Spain, and (c) PYC: Brazil. The main elements that individually contributed to the increase in TTHQ, considering the type of CMF per country, were _i_As, Cd, Co, Cu, Fe, methyl Hg, Ni, Se, and Zn (THQ > 1).

A wide variety of element concentrations was found according to the types of dairy products and the regions identified in this study. In addition, the potential risk of intoxication in babies and children due to some PTE was observed in this study. To sum up, continuous monitoring of the PTE in these products is extremely recommended in order to reduce children’s exposure to non-carcinogenic risk in the respective countries, especially for the elements that showed THQ and TTHQ above 1 in this work.

## Supplementary Information

Below is the link to the electronic supplementary material.

Supplementary Material 1

## Data Availability

No datasets were generated or analysed during the current study.
