# Supplementary material for: Essential and Potentially Toxic Elements in Commercial Milk Formulas: Health Risk Assessment Through a Systematic Review and Meta-analysis
Source: Biol Trace Elem Res. 2026 Apr 9;204(8):6007–28. doi: 10.1007/s12011-026-05088-4 (PMC13368977; doi:10.1007/s12011-026-05088-4)
Supplement: Supplementary file 1 — Supplementary Material 1 [file 12011_2026_5088_MOESM1_ESM.docx]

**Table S1.** PRISMA checklist

| **Section and Topic** | **Item #** | **Checklist item** | **Location where item is reported** |
| --- | --- | --- | --- |
| **TITLE** | | |  |
| Title | 1 | Identify the report as a systematic review. | Page 1 |
| **ABSTRACT** | | |  |
| Abstract | 2 | See the PRISMA 2020 for Abstracts checklist. | Page 2 |
| **INTRODUCTION** | | |  |
| Rationale | 3 | Describe the rationale for the review in the context of existing knowledge. | Page 3 |
| Objectives | 4 | Provide an explicit statement of the objective(s) or question(s) the review addresses. | Page 5 |
| **METHODS** | | |  |
| Eligibility criteria | 5 | Specify the inclusion and exclusion criteria for the review and how studies were grouped for the syntheses. | Page 6-7 |
| Information sources | 6 | Specify all databases, registers, websites, organisations, reference lists, and other sources searched or consulted to identify studies. Specify the date when each source was last searched or consulted. | Page 7-8 |
| Search strategy | 7 | Present the full search strategies for all databases, registers, and websites, including any filters and limits used. | Table S3. Supplementary Material |
| Selection process | 8 | Specify the methods used to decide whether a study met the inclusion criteria of the review, including how many reviewers screened each record and each report retrieved, whether they worked independently, and, if applicable, details of automation tools used in the process. | Page 8 |
| Data collection process | 9 | Specify the methods used to collect data from reports, including how many reviewers collected data from each report, whether they worked independently, any processes for obtaining or confirming data from study investigators, and, if applicable, details of automation tools used in the process. | Page 8 |
| Data items | 10a | List and define all outcomes for which data were sought. Specify whether all results that were compatible with each outcome domain in each study were sought (e.g. for all measures, time points, analyses), and if not, the methods used to decide which results to collect. | Page 9-10 |
|  | 10b | List and define all other variables for which data were sought (e.g. participant and intervention characteristics, funding sources). Describe any assumptions made about any missing or unclear information. | Page 9-10 |
| Study risk of bias assessment | 11 | Specify the methods used to assess risk of bias in the included studies, including details of the tool(s) used, how many reviewers assessed each study, and whether they worked independently, and if applicable, details of automation tools used in the process. | Page 7-8 |
| Effect measures | 12 | Specify for each outcome the effect measure(s) (e.g., risk ratio, mean difference) used in the synthesis or presentation of results. | Page 10-11 |
| Synthesis methods | 13a | Describe the processes used to decide which studies were eligible for each synthesis (e.g., tabulating the study intervention characteristics and comparing against the planned groups for each synthesis (item #5)). | Figure 1 |
|  | 13b | Describe any methods required to prepare the data for presentation or synthesis, such as handling of missing summary statistics or data conversions. | Page 11 |
|  | 13c | Describe any methods used to tabulate or visually display the results of individual studies and syntheses. | Page 11 |
|  | 13d | Describe any methods used to synthesize results and provide a rationale for the choice(s). If meta-analysis was performed, describe the model(s), method(s) to identify the presence and extent of statistical heterogeneity, and software package(s) used. | Page 10-11 |
|  | 13e | Describe any methods used to explore possible causes of heterogeneity among study results (e.g., subgroup analysis, meta-regression). | Page 9 |
|  | 13f | Describe any sensitivity analyses conducted to assess the robustness of the synthesized results. |  |
| Reporting bias assessment | 14 | Describe any methods used to assess the risk of bias due to missing results in a synthesis (arising from reporting biases). | - |
| Certainty assessment | 15 | Describe any methods used to assess certainty (or confidence) in the body of evidence for an outcome. | - |
| **RESULTS** | | |  |
| Study selection | 16a | Describe the results of the search and selection process, from the number of records identified in the search to the number of studies included in the review, ideally using a flow diagram. | Figure 1 |
|  | 16b | Cite studies that might appear to meet the inclusion criteria, but which were excluded, and explain why they were excluded. | Figure 1 |
| Study characteristics | 17 | Cite each included study and present its characteristics. | Table 2 |
| Risk of bias in studies | 18 | Present assessments of risk of bias for each included study. | Figure S1 |
| Results of individual studies | 19 | For all outcomes, present, for each study: (a) summary statistics for each group (where appropriate) and (b) an effect estimate and its precision (e.g., confidence/credible interval), ideally using structured tables or plots. | Table 2 |
| Results of syntheses | 20a | For each synthesis, briefly summarize the characteristics and risk of bias among contributing studies. | - |
|  | 20b | Present the results of all statistical syntheses conducted. If meta-analysis was done, present, for each, the summary estimate and its precision (e.g., confidence/credible interval) and measures of statistical heterogeneity. If comparing groups, describe the direction of the effect. | Page 12-13/Table 2, 4 |
|  | 20c | Present the results of all investigations of possible causes of heterogeneity among study results. | Page 18/Table 2 |
|  | 20d | Present the results of all sensitivity analyses conducted to assess the robustness of the synthesized results. | Page 13, 14 |
| Reporting biases | 21 | Present assessments of risk of bias due to missing results (arising from reporting biases) for each synthesis assessed. | - |
| Certainty of evidence | 22 | Present assessments of certainty (or confidence) in the body of evidence for each outcome assessed. | Table 2 |
| **DISCUSSION** | | |  |
| Discussion | 23a | Provide a general interpretation of the results in the context of other evidence. | Page 14-20 |
|  | 23b | Discuss any limitations of the evidence included in the review. | Page 18 |
|  | 23c | Discuss any limitations of the review processes used. | Page 18 |
|  | 23d | Discuss implications of the results for practice, policy, and future research. | Page 19 |
| **OTHER INFORMATION** | | |  |
| Registration and protocol | 24a | Provide registration information for the review, including the register name and registration number, or state that the review was not registered. | Page 6 |
|  | 24b | Indicate where the review protocol can be accessed, or state that a protocol was not prepared. | Page 6 |
|  | 24c | Describe and explain any amendments to information provided at registration or in the protocol. | Page 6 |
| Support | 25 | Describe sources of financial or non-financial support for the review, and the role of the funders or sponsors in the review. | Page 22 |
| Competing interests | 26 | Declare any competing interests of review authors. | Page 22 |
| Availability of data, code, and other materials | 27 | Report which of the following are publicly available and where they can be found: template data collection forms; data extracted from included studies; data used for all analyses; analytic code; any other materials used in the review. | Page 1 |

**Table S2.** Groups of search terms (PECO strategy) used for the search strategy.

| **PECO’s criteria** | **Descriptions and search terms used for each criterion** |
| --- | --- |
| **Population** | Infant formula (starter and follow-on) and growing-up milks (which includes: growing-up formula, fortified whole milk, growing-up milk) ("*growing up milk*" OR "*young child formula*" OR "*growth formula*" OR "*growing up formula*" OR "*follow up formula*" OR "*follow on formula*" OR "*follow up milk*" OR "*follow on milk*" OR "*toddler milk*" OR "*toddler formula*" OR "*transition formula*" OR "*infant formula*" OR "*breast-milk substitutes*" OR "*milk compounds*" OR "*starter infant formula*" OR "*dairy milk*" OR "*fortified milk*" OR "*enriched milk*") |
| **Exposure** | Potentially toxic elements ("*essential elements*" OR "*trace elements*" OR "*toxic elements*" OR "*heavy metals*" OR "*metalloids*" OR "*trace metals*" OR "*metal toxicity*" OR "*metal poisoning*" OR "*toxic metals*" OR "*metal compounds*" OR "*potentially toxic elements*"*).* |
| **Comparison** | **---** |
| **Outcomes**  **Studies** | Determined concentrations of potentially toxic elements  Cross-sectional studies |

**Legend:** PECO: Population, Exposure, Comparison, and Outcomes.

**Table S3.** Search strategy and date performed in the chosen databases.

| **Database** | Search  (February 20, 2024) |
| --- | --- |
| **PubMed** | **#1** "growing up milk" OR "young child formula" OR "growth formula" OR "growing up formula" OR "follow up formula" OR "follow on formula" OR "follow up milk" OR "follow on milk" OR "toddler milk" OR "toddler formula" OR "transition formula" OR "infant formula" OR "breast-milk substitutes" OR "milk compounds" OR "starter infant formula" OR "dairy milk" OR "fortified milk" OR "enriched milk"  **#2** "essential elements" OR "trace elements" OR "toxic elements" OR "heavy metals" OR "metalloids" OR "trace metals" OR "metal toxicity" OR "metal poisoning" OR "toxic metals" OR "metal compounds" OR "potentially toxic elements"  **#3** ("growing up milk" OR "young child formula" OR "growth formula" OR "growing up formula" OR "follow up formula" OR "follow on formula" OR "follow up milk" OR "follow on milk" OR "toddler milk" OR "toddler formula" OR "transition formula" OR "infant formula" OR "breast-milk substitutes" OR "milk compounds" OR "starter infant formula" OR "dairy milk" OR "fortified milk" OR "enriched milk") AND ("essential elements" OR "trace elements" OR "toxic elements" OR "heavy metals" OR "metalloids" OR "trace metals" OR "metal toxicity" OR "metal poisoning" OR "toxic metals" OR "metal compounds" OR "potentially toxic elements")  #4: #1 AND #2 AND #3 |
| **Embase** | ('growing up milk' OR 'young child formula' OR 'growth formula' OR 'growing up formula' OR 'follow up formula' OR 'follow on formula' OR 'follow up milk' OR 'follow on milk' OR 'toddler milk' OR 'toddler formula' OR 'transition formula' OR 'infant formula'/exp OR 'breast-milk substitutes' OR 'milk compounds' OR 'starter infant formula' OR 'dairy milk' OR 'fortified milk' OR 'enriched milk') AND ('essential elements' OR 'trace elements'/exp OR 'toxic elements' OR 'heavy metals'/exp OR 'metalloids'/exp OR 'trace metals' OR 'metal toxicity' OR 'metal poisoning' OR 'toxic metals' OR 'metal compounds' OR 'potentially toxic elements') |
| **Lilacs** | "milk compounds" OR "infant formula" OR "fortified milk" OR "breast-milk substitutes" and "essential elements" OR "heavy metals" OR "potentially toxic elements" OR "trace elements" |
| **Web of Science** | TS=("growing up milk" OR "young child formula" OR "growth formula" OR "growing up formula" OR "follow up formula" OR "follow on formula" OR "follow up milk" OR "follow on milk" OR "toddler milk" OR "toddler formula" OR "transition formula" OR "infant formula" OR "breast-milk substitutes" OR "milk compounds" OR "starter infant formula" OR "dairy milk" OR "fortified milk" OR "enriched milk") AND TS=("essential elements" OR "trace elements" OR "toxic elements" OR "heavy metals" OR "metalloids" OR "trace metals" OR "metal toxicity" OR "metal poisoning" OR "toxic metals" OR "metal compounds" OR "potentially toxic elements" )  Limits: Topic |
| **Scopus** | ( TITLE-ABS-KEY ( "growing up milk" OR "young child formula" OR "growth formula" OR "growing up formula" OR "follow up formula" OR "follow on formula" OR "follow up milk" OR "follow on milk" OR "toddler milk" OR "toddler formula" OR "transition formula" OR "infant formula" OR "breast-milk substitutes" OR "milk compounds" OR "starter infant formula" OR "dairy milk" OR "fortified milk" OR "enriched milk" ) AND TITLE-ABS-KEY ( "essential elements" OR "trace elements" OR "toxic elements" OR "heavy metals" OR "metalloids" OR "trace metals" OR "metal toxicity" OR "metal poisoning" OR "toxic metals" OR "metal compounds" OR "potentially toxic elements" ) )  Limits: Title, abstract and keywords |
| **Livivo** | KW=("growing up milk" or "young child formula" or "growth formula" or "growing up formula" or "follow up formula" or "follow on formula" or "follow up milk" or "follow on milk" or "toddler milk" or "toddler formula" or "transition formula" or "infant formula" or "breast-milk substitutes" or "milk compounds" or "starter infant formula" or "dairy milk" or "fortified milk" or "enriched milk") AND KW=("essential elements" or "trace elements" or "toxic elements" or "heavy metals" or "metalloids" or "trace metals" or "metal toxicity" or "metal poisoning" or "toxic metals" or "metal compounds" or "potentially toxic elements")  Limits: by keywords |

**Table S4.** Assessment of commercial milk formula consumption in relation to Dietary Reference Intakes.

| **Element** | **Adequate Intake (AI)** | | | | **Tolerable upper intake level (UL)** | | | |
| --- | --- | --- | --- | --- | --- | --- | --- | --- |
|  | **IF**  **(0-3m)** | **IF**  **(3-6m)** | **FFI** | **PYC** | **IF**  **(0-3m)** | **IF**  **(3-6m)** | **FFI** | **PYC** |
| Cr | 132.6 | 204.05 | 6.09 | - | - | - | - | - |
| Cu | 0.99 | 1.53 | 0.62 | - | - | - | - | - |
| Fe | 13.45 | 20.69 | 0.2 | 0.09 | 0.09 | 0.13 | 0.07 | 0.01 |
| Mn | 55.3 | 85.12 | 0.17 | 0.04 | - | - | - | 0.025 |
| Se | 1.27 | 1.96 | 1.17 | 0.07 | 0.42 | 0.65 | 0.39 | 0.02 |
| Zn | 1.42 | 2.18 | 0.72 | - | 0.71 | 1.09 | 0.43 | - |

Values above 1 mean that intake of the element is above the Dietary Reference Intakes. IF: infant formula; FFI: follow-up formulas for older infants; PYC: products for young children; m: months old.

**Table S5.** Mean concentration of by type of commercial milk formula

| **Element** | **IF** | **FFI** | **PYC** |
| --- | --- | --- | --- |
|  | **mg 100 kcal^-1^** | | |
| Al | 0.02 | 0.02 | 0.04 |
| As** | 0.02 | 0.01 | 0.02 |
| Cd** | 0.004 | 0.003 | 0.01 |
| Co | 0.0002 | 0.0002 | 0.00002 |
| Cr | 0.01 | 0.01 | 0.00 |
| Cu* | 38.18 | 40.47 | - |
| Fe | 0.70 | 0.94 | 0.19 |
| Methyl Hg | 0.0002 | 0.00001 | 0.000003 |
| Mn* | 31.77 | 31.00 | 13.82 |
| Ni | 0.0027 | 0.01 | 0.03 |
| Pb** | 0.04 | 0.05 | 0.02 |
| Se* | 3.66 | 7.00 | 0.43 |
| U | 0.0001 | 0.0001 | 0.0001 |
| Zn | 0.54 | 0.65 | - |

*μg/100 kcal; **mg/kg wet weight. IF: infant formula; FFI: follow-up formulas for older infants; PYC: products for young children.

**Table S6.** Meta-analysis of essential and PTE concentrations (μg kg^-1^) in infant formula, according to countries.

| **Elements** | **Country** | ***N* of samples in studies** | **WMD *(95% CI)*** | **Heterogeneity** | |
| --- | --- | --- | --- | --- | --- |
|  |  |  |  | ***p* value** | ***I^2^* (%)** |
| **Al** | Brazil | 5 | 570.21 (439.42-701.00) | <0.001 | 96.28 |
|  | Turkey | 1 | 1290.00 (880.94-1699.06) | <0.001 | - |
|  | Jordan | 1 | 4600.00 (3889.87-5310.13) | <0.001 | - |
|  | France | 1 | 196.00 (146.00-246.00) | <0.001 | - |
|  | Spain | 2 | 303.18 (0-949.16) | 0.056 | 72.53 |
| **As_in_** | Brazil | 3 | 22.41 (13.43-31.39) | <0.001 | 97.82 |
|  | Turkey | 1 | 42.97 (10.91-75.04) | <0.001 | - |
|  | Jordan | 1 | 4.70 (3.98-5.43) | <0.001 | - |
|  | France | 1 | 1.61 (1.36-1.87) | <0.001 | - |
|  | Kuwait | 3 | 9.21 (5.69-12.73) | <0.001 | 96.65 |
|  | Spain | 1 | 0.49 (0.41-0.57) | <0.001 | - |
| **Cd** | Brazil | 5 | 4.51 (3.98-5.03) | 0.139 | 43.21 |
|  | Turkey | 1 | 2.19 (0.19-4.19) | <0.001 | - |
|  | Iran | 1 | 4.97 (4.50-5.44) | <0.001 | - |
|  | Jordan | 1 | 8.00 (4.96-11.04) | <0.001 | - |
|  | France | 1 | 0.39 (0.31-0.47) | <0.001 | - |
| **Co** | Brazil | 5 | 10.00 (7.74-12.26) | 1.00 | 0.00 |
|  | Italy | 11 | 11.00 (9.82-12.18) | <0.001 | 87.40 |
|  | Jordan | 1 | 55.40 (46.29-64.51) | <0.001 | - |
|  | France | 1 | 0.91 (0.81-1.01) | <0.001 | - |
|  | Spain | 1 | 0.25 (0.17-0.33) | <0.001 | - |
| **Cr** | Brazil | 5 | 246.91 (231.07-262.76) | 0.002 | 80.89 |
|  | Jordan | 1 | 588.00 (516.80-659.21) | - | - |
|  | France | 1 | 20.80 (17.47-24.13) | - | - |
|  | Malta | 1 | 290.00 (257.33-322.67) | - | - |
|  | Spain | 2 | 190.97 (0-560.71) | - | 99.8 |
| **Cu** | Brazil | 10 | 2672.33 (1667.44-3677.22) | <0.001 | 99.99 |
|  | Italy | 11 | 1238.10 (1078.15-1398.05) | <0.001 | 99.89 |
|  | Jordan | 1 | 2420.00 (2225.65-2614.35) | <0.001 | - |
|  | Malta | 1 | 3330.00 (3173.20-3486.80) | <0.001 | - |
|  | Spain | 2 | 444.46 (310.94-577.97) | 0.012 | 84.18 |
| **Fe*** | Brazil | 10 | 42.95 (27.57-58.33) | <0.001 | 99.99 |
|  | Turkey | 1 | 61.34 (52.84-69.84) | <0.001 | - |
|  | Italy | 11 | 27.99 (25.48-30.51) | <0.001 | 99.79 |
|  | Jordan | 1 | 65.20 (58.96-71.44) | <0.001 | - |
|  | Malta | 1 | 18.34 (16.70-19.98) | <0.001 | - |
|  | Spain | 2 | 5.99 (5.45-6.52) | 0.695 | - |
| **methyl Hg** | Turkey | 1 | 0.13 (0.05-0.20) | <0.001 | - |
|  | Spain | 2 | 15.36 (0-43.99) | <0.001 | 99.77 |
| **Mn** | Brazil | 10 | 2016.78 (954.37-3079.19) | <0.001 | 100 |
|  | EUA | 1 | 170.00 (151.97-188.03) | <0.001 | - |
|  | Jordan | 1 | 984.00 (804.78-1163.22) | <0.001 | - |
|  | Malta | 1 | 2130.00 (1862.13-2397.87) | <0.001 | - |
|  | Spain | 2 | 123.58 (67.68-179.47) | 0.011 | 84.58 |
| **Ni** | Jordan | 1 | 344.00 (169.54-518.46) | <0.001 | - |
|  | France | 1 | 25.90 (0-67.63) | <0.001 | - |
|  | Spain | 2 | 628.26 (0-2251.47) | 0.06 | 72.17 |
| **Pb** | Brazil | 2 | 23.93 (4.75-43.11) | 0.093 | 64.62 |
|  | Turkey | 2 | 92.53 (0-225.23) | <0.001 | 99.22 |
|  | Iran | 1 | 12.57 (12.12-13.02) | <0.001 | - |
|  | Jordan | 1 | 64.90 (57.45-72.35) | <0.001 | - |
|  | Spain | 2 | 15.15 (0-44.19) | <0.001 | 99.77 |
| **Se** | Brazil | 5 | 344.52 (298.07-390.98) | <0.001 | 83.47 |
|  | Italy | 11 | 144.37 (126.56-162.17) | <0.001 | 98.76 |
|  | Jordan | 1 | 120.00 (106.56-133.45) | <0.001 | - |
|  | Spain | 2 | 28.58 (7.64-49.53) | <0.001 | 92.64 |
| **U** | Brazil | 3 | 8.36 (4.92-11.80) | <0.001 | 98.9 |
|  | Italy | 11 | 2.04 (0.91-3.17) | <0.001 | 99.99 |
|  | Jordan | 1 | 3.02 (2.63-3.41) | <0.001 | - |
|  | Spain | 1 | 0.56 (0.38-0.74) | <0.001 | - |
| **Zn*** | Brazil | 10 | 30.33 (19.20-41.48) | <0.001 | 100 |
|  | Turkey | 1 | 29.72 (27.21-32.23) | <0.001 | - |
|  | Jordan | 1 | 32.90 (29.35-36.45) | <0.001 | - |
|  | Malta | 1 | 27.24 (25.44-29.04) | <0.001 | - |
|  | Spain | 2 | 4.88 (4.31-5.47) | 0.18 | 44.4 |

*Concentration in mg kg^-1^; WMD: weighted mean difference; CI: confidence interval; PTE: potentially toxic elements.

**Table S7.** Meta-analysis of essential and PTE concentrations (μg kg^-1^) in follow-up formulas for older infants, according to countries.

| **Elements** | **Country** | ***N* of samples in studies** | **WMD (95% CI)** | **Heterogeneity** | |
| --- | --- | --- | --- | --- | --- |
|  |  |  |  | ***p* value** | ***I^2^* (%)** |
| **Al** | Brazil | 4 | 838.25 (516.68-1159.82) | <0.001 | 98.46 |
|  | Turkey | 1 | 1540.00 (1323.07-1756.93) | <0.001 | - |
|  | France | 1 | 276.00 (198.35-353.65) | <0.001 | - |
|  | Spain | 1 | 47.07 (31.57-62.57) | <0.001 | - |
| **As_in_** | Brazil | 5 | 21.25 (13.70-28.79) | <0.001 | 95.92 |
|  | Turkey | 1 | 17.50 (0-36.18) | <0.001 | - |
|  | France | 1 | 1.68 (1.48-1.88) | <0.001 | - |
|  | Kuwait | 4 | 7.17 (6.52-7.82) | 0.13 | 44.71 |
|  | Spain | 1 | 1.39 (0.15-2.63) | <0.001 | - |
| **Cd** | Brazil | 4 | 4.92 (3.07-6.78) | <0.001 | 93.66 |
|  | Turkey | 1 | 0.08 (0-0.18) | <0.001 | - |
|  | France | 1 | 0.43 (0.31-0.55) | <0.001 | - |
| **Co** | Brazil | 5 | 10.00 (7.74-12.26) | 1.00 | 0.00 |
|  | Spain | 1 | 0.25 (0.19-0.31) | <0.001 | - |
| **Cr** | Brazil | 5 | 340.18 (295.69-384.68) | <0.001 | 92.81 |
|  | France | 1 | 22.10 (17.55-26.65) | <0.001 | - |
|  | Malta | 1 | 240.00 (220.40-259.60) | <0.001 | - |
|  | Spain | 2 | 200.42 (0-590.10) | <0.001 | 99.62 |
| **Cu** | Brazil | 9 | 2127.23 (1262.00-2992.46) | <0.001 | 99.99 |
|  | Malta | 1 | 3370.00 (3232.80-3507.20) | <0.001 | - |
|  | Spain | 2 | 426.19 (270.33-582.04) | 0.001 | 91.62 |
| **Fe*** | Brazil | 9 | 52.94 (32.08-73.80) | <0.001 | 100 |
|  | Turkey | 1 | 72.60 (66.99-78.21) | <0.001 | - |
|  | Malta | 1 | 18.87 (16.87-20.87) | <0.001 | - |
|  | Spain | 2 | 9.01 (8.63-9.34) | 0.258 | 21.88 |
| **methyl Hg** | Turkey | 1 | 0.06 (0.02-0.10) | <0.001 | - |
|  | Spain | 1 | 0.75 (0.46-1.04) | <0.001 | - |
| **Mn** | Brazil | 9 | 1853.30 (935.79-2770.81) | <0.001 | 100 |
|  | Malta | 1 | 2050.00 (1912.80-2187.20) | <0.001 | - |
|  | Spain | 2 | 102.51 (11.74-193.27) | <0.001 | 95.78 |
|  | Saudi Arabia | 1 | 459.00 (413.74-504.26) | <0.001 | - |
| **Ni** | France | 1 | 26.50 (24.50-28.50) | <0.001 | - |
|  | Malta | 1 | 820.00 (780.80-859.20) | <0.001 | - |
|  | Spain | 2 | 398.14 (0-1207.12) | <0.001 | 95.48 |
| **Pb** | Brazil | 2 | 13.98 (3.82-24.14) | 0.16 | 49.54 |
|  | Turkey | 2 | 110.98 (0-243.55) | <0.001 | 95.03 |
|  | Spain | 2 | 15.12 (0-44.17) | <0.001 | 99.59 |
|  | Saudi Arabia | 1 | 57.00 (53.61-60.39) | <0.001 | - |
|  | Ethiopia | 4 | 35.43 (0.04-70.81) | 0.46 | 10.54 |
| **Se** | Brazil | 5 | 462.50 (223.98-701.02) | <0.001 | 99.32 |
|  | Spain | 2 | 22.51 (10.45-34.56) | 0.03 | 78.64 |
| **U** | Brazil | 5 | 7.22 (2.49-11.95) | <0.001 | 99.37 |
|  | Spain | 1 | 0.70 (0.35-1.05) | <0.001 | - |
| **Zn*** | Brazil | 9 | 28.59 (16.59-40.59) | <0.001 | 100 |
|  | Turkey | 1 | 34.64 (30.72-38.56) | <0.001 | - |
|  | Malta | 1 | 33.00 (32.37-33.63) | <0.001 | - |
|  | Spain | 2 | 5.66 (4.02-7.31) | 0.021 | 81.16 |
|  | Ethiopia | 5 | 45.43 (31.35-59.50) | <0.001 | 99.89 |

*Concentration in mg kg^-1^; WMD: weighted mean difference; CI: confidence interval; PTE: potentially toxic elements.

**Table S8.** Meta-analysis of essential and PTE concentrations (μg kg^-1^) in products for young children, according to the country.

| **Elements** | **Country** | ***N* of samples in studies** | **WMD (95% CI)** | **Heterogeneity** | |
| --- | --- | --- | --- | --- | --- |
|  |  |  |  | ***p* value** | ***I^2^* (%)** |
| **Al** | Brazil | 6 | 1522.47 (184.70-2860.23) | <0.001 | 99.79 |
|  | France | 1 | 189.00 (174.63-203.37) | <0.001 | - |
|  | Spain | 1 | 380.00 (175.24-584.76) | <0.001 | - |
|  | Saudi Arabia | 1 | 8690.00 (8169.47-9210.53) | <0.001 | - |
| **As_in_** | Brazil | 6 | 40.03 (25.79-54.27) | <0.001 | 98.92 |
|  | Turkey | 1 | 0.96 (0-1.94) | <0.001 | - |
|  | France | 1 | 2.11 (1.60-2.62) | <0.001 | - |
|  | Kuwait | 3 | 7.40 (5.55-9.24) | <0.001 | 89.31 |
| **Cd** | Brazil | 6 | 8.24 (6.12-10.37) | <0.001 | 95.54 |
|  | Turkey | 1 | 1.30 (0.50-2.10) | <0.001 | - |
|  | France | 1 | 0.71 (0-1.51) | <0.001 | - |
| **Co** | France | 1 | 0.90 (0.80-1.00) | <0.001 | - |
| **Cr** | France | 1 | 27.70 (16.39-39.01) | <0.001 | - |
| **Fe*** | Spain | 1 | 8.89 (6.58-11.21) | <0.001 | - |
| **methyl Hg** | Turkey | 1 | 0.14 (0-0.30) | <0.001 | - |
| **Mn** | Spain | 1 | 140.00 (94.04-185.96) | <0.001 | - |
|  | Saudi Arabia | 1 | 1160.00 (1103.42-1216.58) | - | - |
| **Ni** | Brazil | 6 | 1490.97 (856.54-2125.39) | <0.001 | 99.83 |
|  | Spain | 1 | 550.00 (341.07-758.93) | <0.001 | - |
| **Pb** | Brazil | 5 | 5.36 (2.53-8.20) | <0.001 | 90.67 |
|  | Turkey | 1 | 6.96 (5.33-8.59) | <0.001 | - |
|  | Saudi Arabia | 1 | 79.00 (75.49-82.51) | <0.001 | - |
| **Se** | Spain | 1 | 20.00 (11.65-28.35) | <0.001 | - |
| **U** | Brazil | 6 | 6.65 (4.2-9.11) | <0.001 | 89.28 |

*Concentration in mg kg^-1^; WMD: weighted mean difference; CI: confidence interval; PTE: potentially toxic elements.

**Table S9.** THQ of toxic elements in commercial milk formulas for children between 0 and three years, according to different countries.

| **Country** | **CMF** | **Al** | **As** | **Cd** | **Co** | **Cr** | **Cu** | **Fe** | **Hg** | **Mn** | **Ni** | **Se** | **U** | **Zn** |
| --- | --- | --- | --- | --- | --- | --- | --- | --- | --- | --- | --- | --- | --- | --- |
|  |  | **mean (minimum-maximum)** | | | | | | | | | | | | |
| Brazil | IF | 0.012 (0.009-0.014) | 1.532 (0.918-2.146) | 0.925 (0.816-1.032) | 0.684 (0.529-0.838) | 0.003(0.003-0.004) | 1.37 (0.855-1.885) | 1.258 (0.808-1.709) | - | 0.295 (0.14-0.451) | - | 1.413 (1.223-1.604) | 0.857 (0.505-1.21) | 2.073 (1.313-2.836) |
|  | FFI | 0.013 (0.008-0.017) | 1.066 (0.688-1.445) | 0.741 (0.462-1.021) | 0.502 (0.388-0.615) | 0.003 (0.003-0.004) | 0.801 (0.475-1.126) | 1.139 (0.69-1.587) | - | 0.199 (0.101-0.298) | - | 1.393 (0.674-2.111) | 0.543 (0.187-0.9) | 1.435 (0.833-2.037) |
|  | PYC | 0.009 (0.001-0.018) | 0.827 (0.533-0.501) | 0.511 (0.379-0.643) | - | - | - | - | - | - | 0.84 (0.483-1.198) | - | 0.206 (0.13-0.282) | - |
| Ethiopia | FFI | - | - | - | - | - | - | - | - | - | - | - | - | 2.28 (1.573-2.986) |
| Iran | IF | - | - | 1.019 (0.923-1.116) | - | - | 0 (0-0) | - | - | - | - | - | - | - |
| Italy | IF | - | - | - | 0.752 (0.671-0.833) | - | 0.635 (0.553-0.717) | 0.82 (0.746-0.894) | - | - | - | 0.592 (0.519-0.665) | 0.209 (0.093-0.325) | - |
| Jordan | IF | 0.094 (0.08-0.109) | 0.321 (0.272-0.371) | 1.641 (1.017-2.264) | 3.787 (3.164-4.41) | 0.008 (0.007-0.009) | 1.241 (1.141-1.34) | 1.91 (1.727-2.093) | - | 0.144 (0.118-0.17) | 0.641 (0.316-0.967) | 0.492 (0.437-0.547) | 0.31 (0.27-0.35) | 2.249 (2.006-2.492) |
| France | IF | 0.004 (0.003-0.005) | 0.11 (0.093-0.128) | 0.08 (0.064-0.096) | 0.062 (0.055-0.069) | 0 (0-0) | 0 (0-0) | - | - | - | 0.048 (0-0.126) | - | - | - |
|  | FFI | 0.004 (0.003-0.005) | 0.084 (0.074-0.094) | 0.065 (0.047-0.083) | - | 0 (0-0) | - | - | - | - | 0.036 (0.034-0.039) | - | - | - |
|  | PYC | 0.001 (0.001-0.001) | 0.044 (0.033-0.054) | 0.044 (0-0.094) | 0.019 (0.017-0.021) | 0 (0-0) | - | - | - | - | - | - | - | - |
| Kuwait | IF | - | 0.63 (0.389-0.87) | - | - | - | 0 (0-0) | - | - | - | - | - | - | - |
|  | FFI | - | 0.36 (0.327-0.392) | - | - | - | - | - | - | - | - | - | - | - |
|  | PYC | - | 0.153 (0.115-0.191) | - | - | - | - | - | - | - | - | - | - | - |
| Malta | IF | - | - | - | - | 0.004 (0.004-0.004) | 1.707 (1.627-1.788) | 0.537 (0.489-0.585) | - | 0.312 (0.273-0.351) | - | - | - | 1.862 (1.739-1.985) |
|  | FFI | - | - | - | - | 0.002 (0.002-0.003) | 1.268 (1.217-1.32) | 0.406 (0.363-0.449) | - | 0.22 (0.206-0.235) | 1.122 (1.069-1.176) | - | - | 1.656 (1.624-1.688) |
| Saudi Arabia | FFI | - | - | - | - | - | - | - | - | 0.049 (0.044-0.054) | - | - | - | - |
|  | PYC | 0.054 (0.051-0.057) | - | - | - | - | - | - | - | - | - | - | - | - |
| Turkey | IF | 0.026 (0.018-0.035) | 2.937 (0.746-5.13) | 0.449 (0.039-0.859) | 0.752 (0.671-0.833) | - | 0 (0-0) | 1.797 (1.548-2.046) | 0.027 (0.01-0.041) | - | - | - | - | 2.032 (1.86-2.203) |
|  | FFI | 0.023 (0.02-0.026) | 0.878 (0-1.816) | 0.012 (0-0.027) | - | - | - | 1.561 (1.441-1.682) | 0.009 (0.003-0.015) | - | - | - | - | 1.738 (1.542-1.935) |
|  | PYC | - | 0.02 (0-0.04) | 0.081 (0.031-0.13) | - | - | - | - | 0.009 (0-0.019) | - | - | - | - | - |
| United States | IF | - | - | - | - | - | 0 (0-0) | - | - | 0.025 (0.022-0.028) | - | - | - | - |
| Spain | IF | 0.006 (0-0.019) | 0.033 (0.028-0.039) | - | 0.017 (0.012-0.023) | 0.003 (0-0.008) | 0.228 (0.159-0.296) | 0.175 (0.16-0.191) | 3.15 (0-9.022) | 0.018 (0.01-0.026) | 1.171 (0-4.198) | 0.117 (0.031-0.203) | 0.057 (0.039-0.076) | 0.334 (0.295-0.374) |
|  | FFI | 0.001 (0-0.001) | 0.07 (0.008-0.132) | - | 0.013 (0.01-0.016) | 0.002 (0-0.006) | 0.16 (0.102-0.219) | 0.194 (0.186-0.201) | 0.113 (0.069-0.157) | 0.011 (0.001-0.021) | 0.545 (0-1.652) | 0.068 (0.031-0.104) | 0.053 (0.026-0.079) | 0.284 (0.202-0.367) |
|  | PYC | 0.002 (0.001-0.004) | - | - | - | - | - | 0.079 (0.058-0.099) | - | 0.006 (0.004-0.008) | 0.31 (0.192-0.428) | 0.025 (0.014-0.035) | - | - |

IF: infant formula; FFI: follow-up formulas for older infants; PYC: products for young children.


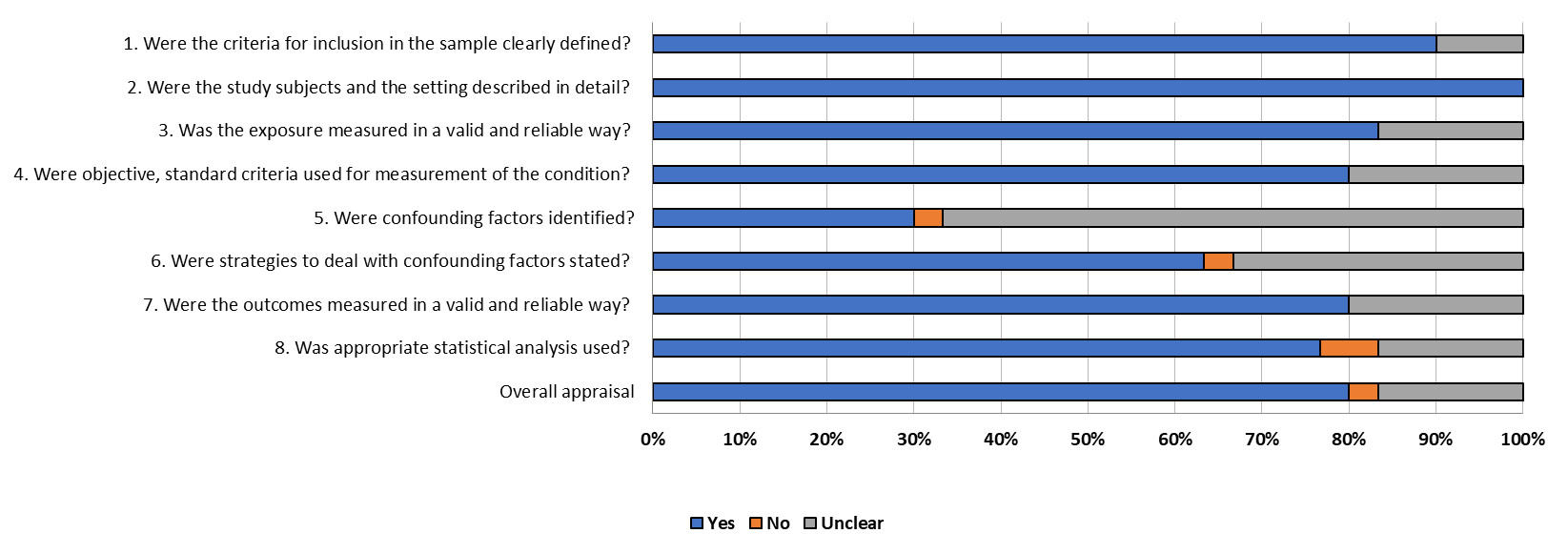


**Figure S1.** Risk of bias summary of the included studies based on the Joanna Briggs Institute Critical Appraisal Checklist for Analytical Cross-Sectional Studies.
